# IL-4 downregulates gap junction protein connexin 26 to promote HIV-1 infection in macrophages

**DOI:** 10.1128/mbio.01626-25

**Published:** 2025-09-22

**Authors:** Shumei Wang, Jingjing Zhang, Yuan Liu, Li Zhao, Yimin Zhang, Guoxin Liang, Hong Shang

**Affiliations:** 1 State Key Laboratory for Diagnosis and Treatment of Infectious Diseases, National Health Commission Key Laboratory of AIDS Prevention and Treatment, National Clinical Research Center for Laboratory Medicine, The First Hospital of China Medical University 159407, Shenyang, China; 2 Center for Cell and Gene Therapy, The First Hospital of China Medical University 159407, Shenyang, China; 3 Key Laboratory of AIDS Immunology, Chinese Academy of Medical Sciences 567925, Shenyang, China; 4 State Key Laboratory for Diagnosis and Treatment of Infectious Diseases, National Clinical Research Center for Infectious Diseases, Collaborative Innovation Center for Diagnosis and Treatment of Infectious Diseases, The First Affiliated Hospital, College of Medicine, Zhejiang University 12377, Hangzhou, China; 5 Collaborative Innovation Center for Diagnosis and Treatment of Infectious Diseases, Hangzhou, China; Duke University School of Medicine, Durham, North Carolina, USA

**Keywords:** HIV-1, IL-4, GJB2

## Abstract

Macrophages and dendritic cells (DCs) are important targets for HIV-1 replication *in vivo*. Myeloid cells are collectively more resistant to HIV-1 infection than CD4^+^ T lymphocytes, but interleukin (IL)-4 has been observed to promote macrophage susceptibility to HIV-1 infection. However, the mechanism remains unclear. Herein, we found that IL-4 enhanced HIV-1 infection in myeloid lineage macrophages by downregulating the novel antiviral factor gap junction protein beta 2 (GJB2) located on the cell membrane. In the absence of GJB2, the IL-4-mediated enhancement of HIV-1 replication was largely impaired in primary macrophages. Conversely, IL-6-mediated enhancement of HIV-1 infection was unaffected by GJB2 depletion. GJB2 was constitutively expressed in myeloid cells but not in CD4^+^ T cells, and GJB2 silencing enhanced HIV-1 transmission in macrophages and DCs. Additionally, GJB2 required Ca2^+^ to exert its antiviral activity by interfering with HIV-1 attachment to target cells. Because IL-4 downregulated GJB2 expression to enhance HIV-1 infection in macrophages, GJB2 may be a novel target for the treatment of HIV-1 infection.

**IMPORTANCE:**

HIV-1 primarily targets two groups of cells *in vivo*: CD4^+^ T lymphocytes and myeloid lineage cells, such as macrophages and dendritic cells. Although myeloid cells are more resistant to HIV-1 infection than CD4^+^ T cells, some cytokines, including interleukin (IL)-4 and IL-6, promote myeloid cell infection. Gap junction protein beta 2 (GJB2) is particularly relevant in the field of auditory science. Here, we identified GJB2 as a novel antiviral factor by demonstrating that IL-4-mediated reduction in GJB2 levels enhanced HIV-1 infection in myeloid cells. Interestingly, GJB2 expression was regulated by IL-4 but not by interferons. The reduction in GJB2 levels was inversely correlated with increased HIV-1 infection levels, suggesting the potential of GJB2 for combating HIV/AIDS.

## INTRODUCTION

Myeloid lineage cells, such as macrophages and dendritic cells (DCs), are crucial for innate and adaptive immune responses to microorganisms and are important targets of HIV-1 infection *in vivo* (1–9). Macrophages are the first line of defense against HIV-1, becoming differentially activated in response to the microenvironment (10). The classical pathway of interferon (IFN)-γ-dependent activation of M1 macrophages by T helper 1 (Th1)-type responses is a well-established feature of cellular immunity to HIV-1 infection. In the presence of cytokines produced in Th2-type responses, such as interleukin (IL)-4, macrophages become differentially activated into M2 macrophages, which play an important role in HIV-1 pathogenesis (10). The susceptibility of macrophages to HIV-1 infection is influenced by a wide variety of stimuli. Additionally, the differentiation stage of monocytes/macrophages modulates their permissiveness to HIV-1, with primary monocytes/macrophages being less susceptible to HIV-1 than differentiated macrophages (11–14). Investigations of macrophage differentiation or activation effects on HIV-1 replication have provided some insight into the impact of specific microenvironments on macrophage infection *in vivo* (7, 9). Although the cytokine IL-4 exerts potent biological effects on monocytes by reducing their initial adherence to the extracellular matrix (15), increasing major histocompatibility complex class II expression (16), and enhancing the production of both granulocyte and macrophage colony-stimulating factors (M-CSFs) (17), it has received little attention. However, IL-4 has been reported to enhance HIV-1 infection in primary monocytes/macrophages without affecting the expression of CC chemokine receptor 5 (CCR5), an HIV coreceptor (18–20), but the underlying mechanism is unknown. Thus, this investigation aimed to elucidate the mechanism of IL-4-mediated enhancement of HIV-1 infection.

Gap junction protein beta 2 (GJB2) is expressed in the epidermis and cochlea (21) and is particularly relevant in the field of auditory science (22). It belongs to the transmembrane protein family of connexins, and its single coding exon (exon 2) encodes connexin 26 (CX26), a 26-kD isoform of connexin. CX26 forms channels between cells that, when open, allow cell-to-cell diffusion of small molecules. Some mutant alleles of GJB2 cause autosomal dominant nonsyndromic hearing loss through a dominant-negative effect (23, 24). Our study identified GJB2 as a constitutively expressed novel antiviral factor that inhibited HIV-1 entry into the macrophages and DCs. In the absence of GJB2, HIV-1 spread was largely promoted by primary macrophages and DCs. Further evidence suggested that GJB2 required Ca^2+^ to exert its antiviral activity. Interestingly, GJB2 silencing abrogated the IL-4-mediated enhancement of HIV-1 infection. Therefore, IL-4 enhanced HIV-1 infection in myeloid lineage macrophages by downregulating the antiviral factor GJB2. Collectively, our findings suggested that GJB2 was a potential novel target for the treatment of HIV-1 infection.

## RESULTS

### GJB2 inhibits HIV-1 replication in target cells

**Fig 1:**
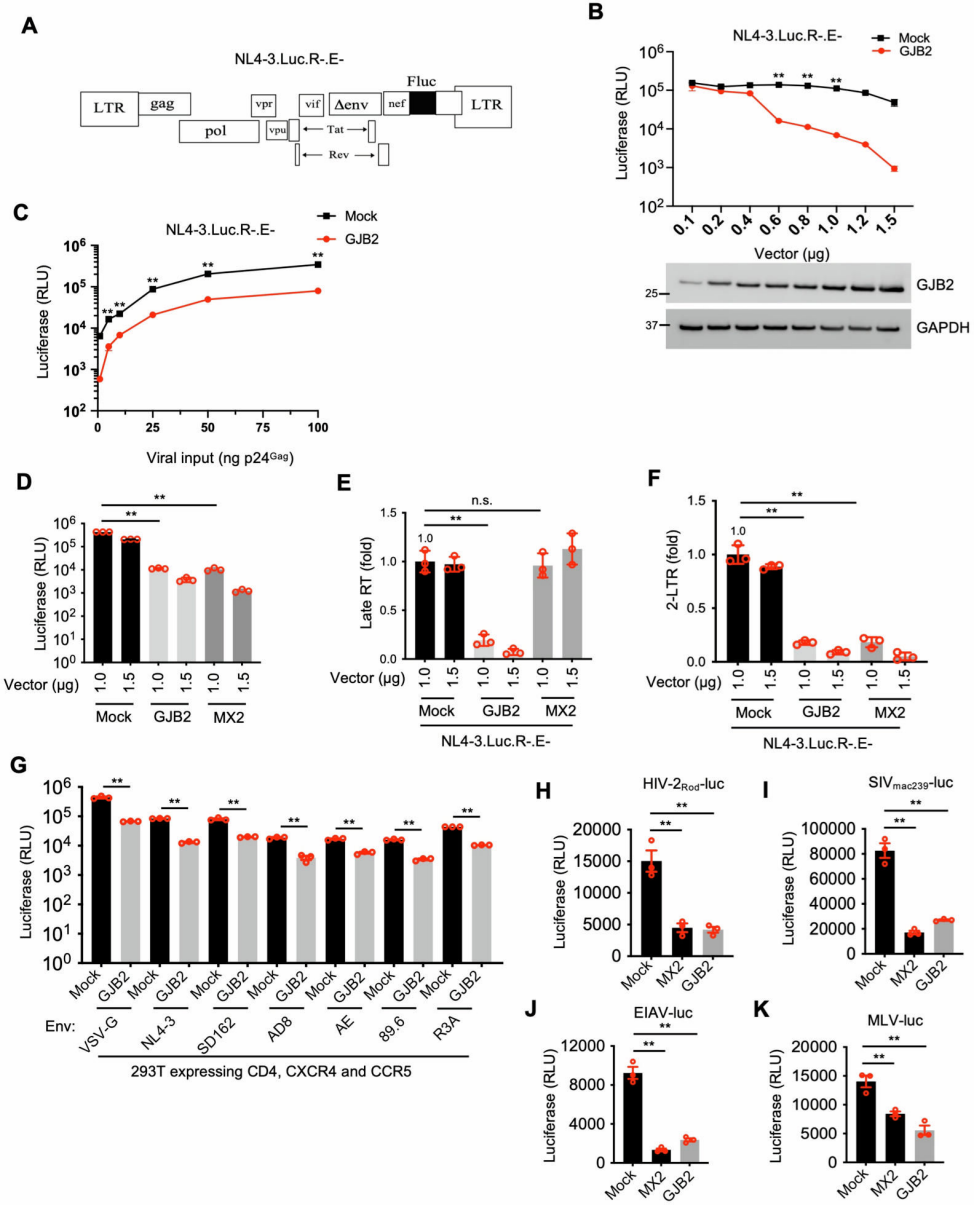
GJB2 inhibits HIV-1 replication in target cells. (**A**) The schematic diagram of the NL4-3.Luc.R-E-. (**B**)293T cells were transfected with a construct encoding non-tagged GJB2 or mock expression constructs at the indicated doses. After 24 h of transfection, the cells were infected with 50 ng p24 of HIV-1_NL4-3.Luc.R−E−_ (VSV-G) and lysed 24 h later to measure luciferase reporter activity. Data are presented as the mean ± SEM from three independent experiments. **P < 0.01 (two-tailed unpaired Student’s *t*-test). Cells were lysed for western blotting to assess GJB2 and GAPDH expression. (**C**) 293T cells were transfected with a construct encoding non-tagged GJB2 or a mock expression construct. After 24 h of transfection, the cells were infected with 1, 5, 10, 25, 50, or 100 ng HIV-1_NL4-3.Luc.R−E−_ (VSV-G) and lysed 24 h later to measure luciferase reporter activity. Data are presented as the mean ± SEM from three independent experiments. **P < 0.01 (two-tailed unpaired Student’s *t*-test). (**D and E**) 293T cells were transfected with a construct encoding FLAG-tagged GJB2, MX2, or mock expression constructs for 24 h. Then the cells were infected with 50 ng HIV-1_NL4-3.Luc.R−E−_ (VSV-G). After 24 h, the cells were lysed to measure luciferase reporter activity (**D**), and total DNA was extracted for qPCR to measure viral late RT (**E**) and circular 2-LTR DNA (**F**). **P < 0.01; n.s., not significant (two-tailed unpaired Student’s *t*-test). Data are presented as the mean ± SEM of three independent experiments. (**G**) GJB2 restricts HIV-1 infection in an Env-independent manner. 293T cells were cotransfected with a construct encoding FLAG-tagged GJB2 or a mock expression construct along with the CD4, CXCR4, and CCR5 expression vectors. After 24 h of transfection, the cells were infected with 50 ng p24 of HIV-1_NL4-3.Luc.R−E−_ reporter virus with VSV-G, CXCR4-, CCR5-, or dual-tropic Env as indicated. After 24 h of infection, the cells were lysed to measure luciferase reporter activity. Data are presented as the mean ± SD from three triplicates. **P < 0.01; n.s., not significant (two-tailed unpaired Student’s *t*-test). (**H–K**) 293T cells were transfected with FLAG-tagged GJB2, MX2, or mock expression constructs. After 24 h of transfection, the cells were infected with VSV-G-pseudotyped HIV-2_Rod_-luc (**H**), SIV_mac239_-luc (**I**), EIAV-luc (**J**), or MLV-luc (**K**) for 48 h and lysed to measure luciferase reporter activity. **P < 0.01; n.s., not significant (two-tailed unpaired Student’s *t*-test). Data are presented as the mean ± SEM of three independent experiments.

GJB2 protein is localized on the cell membrane. Thus, we first overexpressed an untagged GJB2 expression construct in 293T cells and subsequently infected 293T cells with vesicular stomatitis virus G protein (VSV-G)-pseudotyped HIV-1_NL4-3.Luc.R−E−_. Our findings demonstrated that GJB2 restricted this pseudotyped HIV-1 replication in a dose-dependent manner (Fig. 1A and C). Next, we took advantage of MX2, a host restriction factor inhibiting HIV-1 core translocation into the nucleus, to explore the underlying molecular mechanism responsible for GJB2 inhibition of HIV-1 infection. MX2 and GJB2 consistently inhibited HIV-1 infection, and this was statistically significant for GJB2 (Fig. 1D). Our results showed that GJB2 significantly inhibited viral late reverse transcript (late RT) and nuclear two-long terminal repeat DNA circles (2-LTR) circular DNA. In agreement with previous findings (25, 26), MX2 exhibited an obvious inhibition of 2-LTR circular DNA but not viral late RT (Fig. 1E and F). Because viral late RT was significantly reduced by GJB2 expression, this indicated that the molecular mechanism underlying GJB2 inhibition of HIV-1 replication differed from that of MX2. We also investigated the effect of GJB2 on the replication of HIV-1 virions combined with CXCR4-, CCR5-, or dual-tropic envelope (Env) proteins in 293T cells that expressed CD4 receptor and coreceptors CXCR4 and CCR5. We found that GJB2 consistently inhibited HIV-1 replication despite different Env combinations and also inhibited VSV-G (Fig. 1G), indicating that GJB2-mediated inhibition was independent of Env. This led us to question whether HIV-1 accessory proteins or HIV-2 Vpx might compromise this GJB2-mediated inhibition to rescue HIV-1 infection in target cells. Our results showed that Nef, Vif, Vpr, Vpu, and Vpx did not have an obvious effect on the anti-HIV-1 activity of GJB2 (Fig. S1A and D), suggesting that GJB2 was insensitive to these accessory proteins. Next, our goal was to determine whether GJB2 exerted broad antiviral activity by challenging GJB2-overexpressing cells with VSV-G-pseudotyped HIV-2, simian immunodeficiency virus (SIV), equine infectious anemia virus (EIAV), and murine leukemia virus (MLV) reporter (Fig. 1H through K ). We observed that GJB2 inhibited the replication of all of these viruses in the target cells, suggesting that it may exert broad antiviral activity against retroviruses.

### GJB2 is mainly expressed in macrophages and DCs

**Fig 2:**
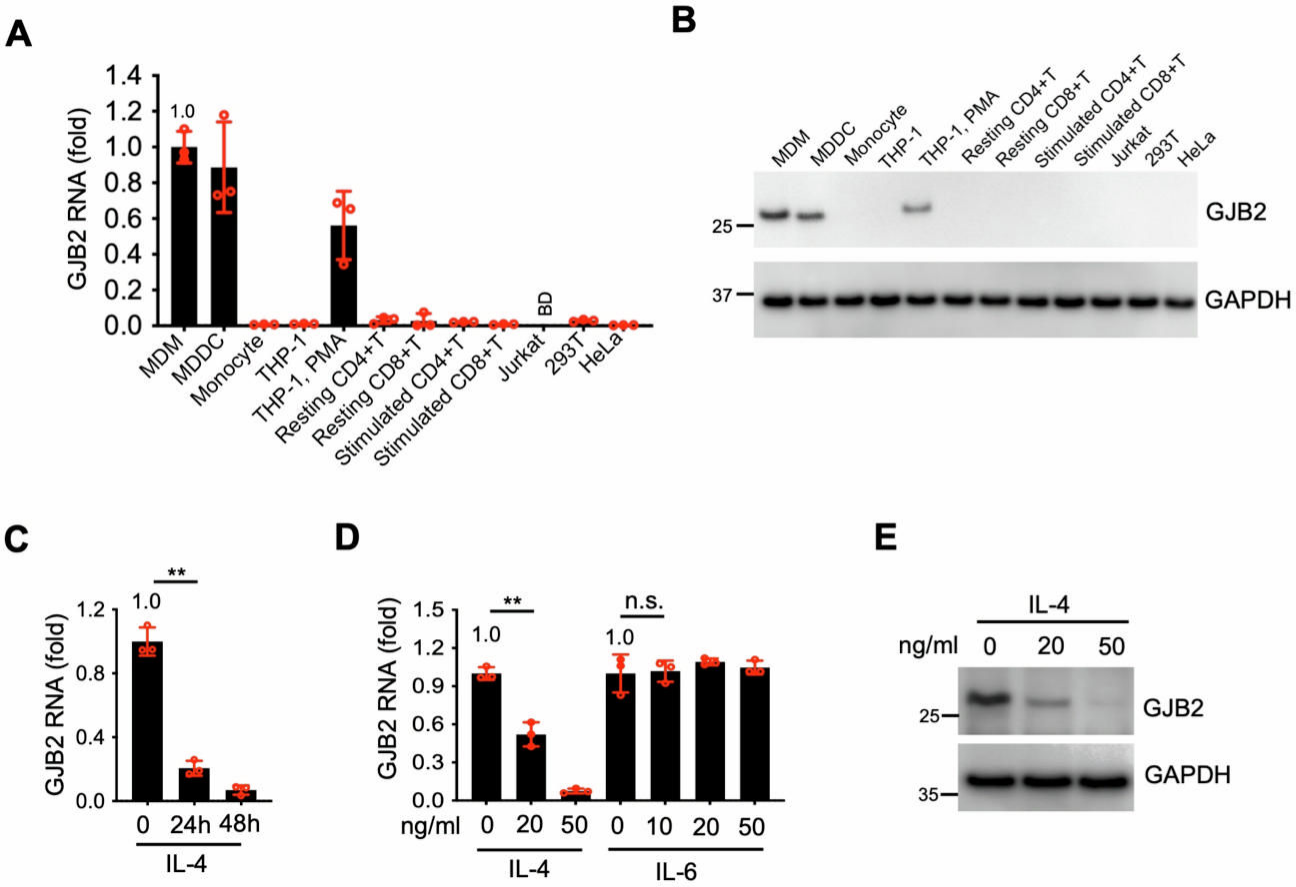
GJB2 expression is downregulated by IL-4 in macrophages. (**A**) Total RNA was extracted from primary monocytes, monocyte-derived macrophages (MDMs), monocyte-derived dendritic cells (MDDCs), THP-1 cells, phorbol-12-myristate 13-acetate (PMA)-treated THP-1 cells, stimulated or resting CD4^+^ and CD8^+^ T cells, and established cell lines (Jurkat, 293T, and HeLa). GJB2 transcript levels were measured using quantitative PCR and normalized against GAPDH levels. BD, below the detection limit. (**B**) Monocytes, MDMs, MDDCs, THP-1 cells, PMA-treated THP-1 cells, stimulated or resting CD4^+^ and CD8^+^ T cells, and established cell lines (Jurkat, 293T, and HeLa) were lysed for western blotting to assess GJB2 and GAPDH protein levels. (**C**) MDMs were treated with or without IL-4 (20 ng/mL) for 24 or 48 h. Total RNA was extracted for qPCR to measure the transcript levels of *GJB2* normalized against the *GAPDH* levels. (**D**) MDMs were treated with or without IL-4 or IL-6 at the indicated doses for 24 h. Total RNA was extracted for qPCR to measure the transcript levels of *GJB2* normalized against the *GAPDH* levels. Data are presented as the mean ± SEM from three independent experiments. **P < 0.01; n.s., not significant (two-tailed unpaired Student’s *t*-test). (**E**) MDMs were treated with or without IL-4 as indicated for 24 h and lysed for western blotting to assess GJB2 and GAPDH protein levels. All western blotting data represent three independent experiments.

We examined the GJB2 expression profile in different target cells, revealing that GJB2 was mainly expressed in differentiated myeloid cells but not in CD4^+^ and CD8^+^ T cells or in Jurkat, 293T, and HeLa cell lines (Fig. 2A and B). We then investigated whether IFNs stimulated GJB2 expression to restrict HIV-1 infection. Our results showed IFN-α, -β, and -γ did not induce GJB2 upregulation in primary CD4^+^ T cells or macrophages (Fig. S2A and S2B). Interestingly, IL-4 decreased GJB2 expression in primary macrophages (Fig. 2C and D). In contrast, GJB2 expression was unaffected by both the presence and absence of cytokine IL-6, which was reported to promote HIV-1 infection in primary macrophages (10, 27). GJB2 expression was reduced in a dose-dependent manner in the presence of IL-4 (Fig. 2D and E). Because GJB2 is a transmembrane protein (21, 22), we first needed to exclude the possibility that the green fluorescent protein (GFP) tagged to its C-terminus influenced its antiviral activity. Our findings revealed that C-terminal GFP-tagged GJB2 (GJB2-GFP) and FLAG-tagged GJB2 exhibited similar anti-HIV activity to non-tagged GJB2 in the target cells (Fig. S3A). Next, we investigated the cellular localization of GJB2 in 293T and primary CD4^+^ T cells using the fusion protein GJB2-GFP. We stained GJB2-expressing viable cells using a GJB2-specific monoclonal antibody to examine the cell membrane localization of GJB2 in 293T cells and stimulated CD4^+^ T cells. In contrast to the isotype control antibody IgG, both the C-terminus of GFP-tagged and non-tagged GJB2 appeared as a distinct membrane-localized pattern in 293T cells and stimulated CD4^+^ T cells (Fig. S3B and S3C). Moreover, GJB2-GFP emitted a distinct fluorescent membrane signal in 293T cells and CD4^+^ T cells (Fig. S3D and S3E). We also examined the localization of endogenous GJB2 in primary macrophages and DCs. We observed the consistent localization of GJB2 on the cell membrane in a manner similar to Na^+^/K^+^-ATPase, which is an integrated plasma membrane protein (Fig. S3F). Moreover, we also purified the membrane-bound proteins to determine the subcellular localization of GJB2 in primary macrophages or DCs. GJB2 was consistently found on the cell membrane just like Na^+^/K^+^-ATPase (Fig. S3G and S3H). Collectively, these findings suggested that GJB2 was synthesized in macrophages and DCs and localized on the cell membrane.

### GJB2 overexpression inhibits HIV-1 spread in primary CD4^+^ T cells

**Fig 3:**
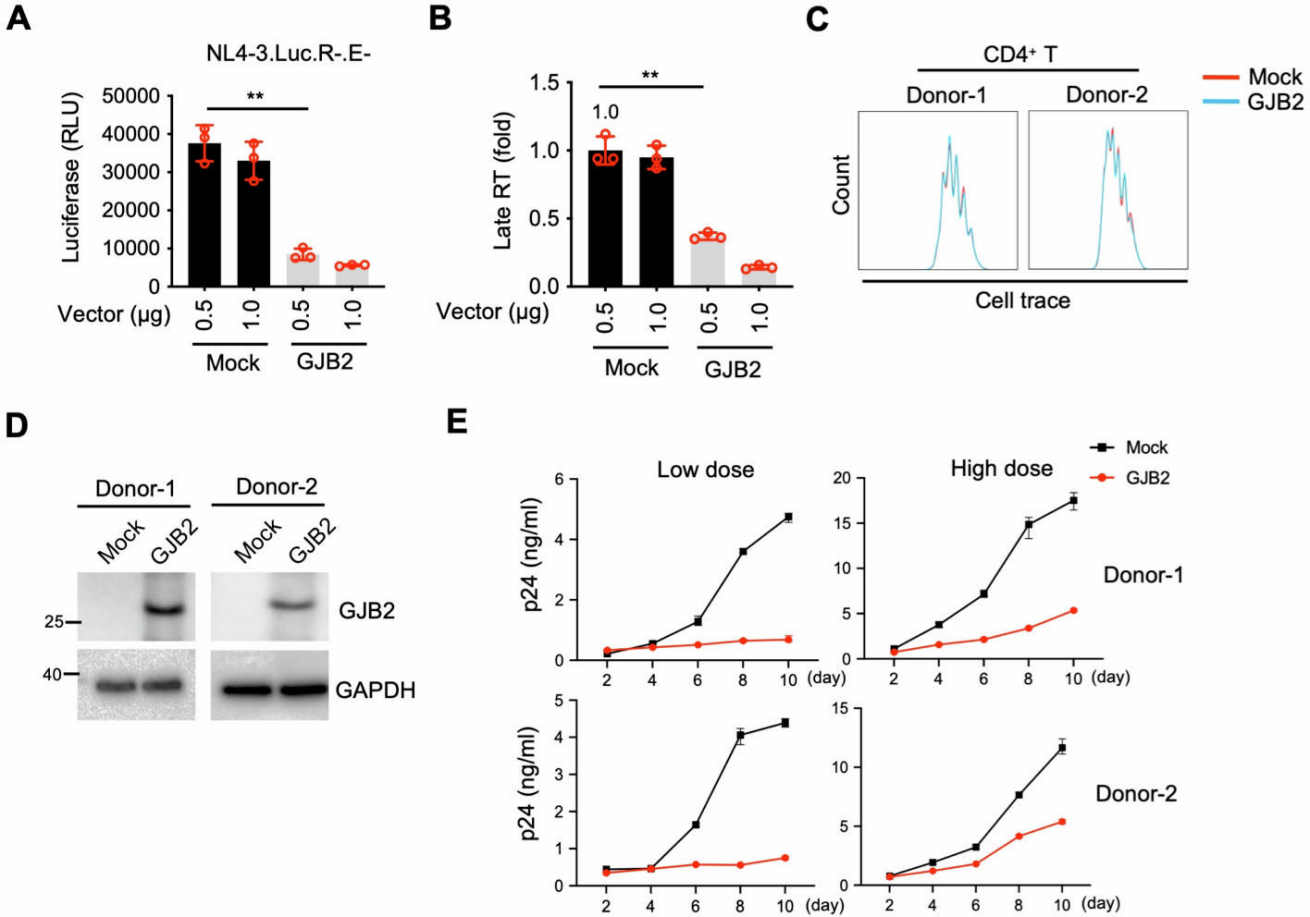
GJB2 inhibits HIV-1 spread in CD4^+^ T cells. (**A and B**) Stimulated cells were electroporated with a construct encoding non-tagged GJB2 or mock expression constructs at the indicated doses. After 24 h of transfection, the cells were infected with 10 ng HIV-1_NL4-3.Luc.R−E−_(VSV-G). After 24 h, the cells were lysed to measure luciferase reporter activity (**A**), and total DNA was extracted for qPCR to measure viral late RT (**B**). Data are presented as the mean ± SEM from three independent experiments. **P < 0.01. (C–E) Lentiviral non-tagged GJB2 or mock (GFP) expression vector-transduced stimulated CD4^+^ T cells were infected with 5 ng (low dose) or 50 ng (high dose) of HIV-1_NL4-3_ for 10 days. The aliquoted cells were stained with CellTrace Violet to assess proliferation (**C**). Cells were lysed for western blotting to assess GJB2 and GAPDH expression (**D**). Viral production in culture was measured by p24 enzyme-linked immunosorbent assay at the indicated time points (**E**). Data are presented as the mean ± SD of triplicates.

Next, to confirm the inhibitory effect of GJB2 on HIV-1 infection in stimulated CD4^+^ T cells, we infected stimulated CD4^+^ T cells with VSV-G-pseudotyped HIV-1. We observed that overexpressed GJB2 restricted HIV-1 replication in a dose-dependent manner (Fig. 3A). GJB2 also significantly inhibited viral late RT (Fig. 3B). To validate this inhibitory effect, we investigated the underlying molecular mechanism of GJB2 inhibition of HIV-1 spread in stimulated CD4^+^ T cells. As a result, GJB2 expression did not noticeably affect primary CD4^+^ T cell proliferation (Fig. 3C and D). However, GJB2 restricted the spread of CXCR4-tropic HIV-1 in dividing CD4^+^ T cells (Fig. 3E), indicating that this inhibitory effect was due to the presence of GJB2. Next, we investigated whether GJB2 inhibited cell-to-cell HIV-1 transmission. CD4^+^ T cells were transduced with lentiviral vectors expressing GJB2 and subsequently stained with CellTrace Violet. These cells were then co-cultured with HIV-1-producing ACH-2 cells. In the absence of a transwell culture system, GJB2 expression reduced the percentage of Gag-positive CD4^+^ T cells from 4.78% to 1.68% (Fig. S4A and B). When transwell inserts were used to prevent direct cell-to-cell contact, GJB2 similarly reduced the proportion of Gag-positive cells from 1.96% to 0.79%. Collectively, these findings indicate that GJB2 suppresses cell-to-cell HIV-1 transmission and thereby inhibits HIV-1 infection in target CD4^+^ T cells.

### GJB2 overexpression inhibits HIV-1 spread in CD4^+^ T cells *ex vivo*

To validate GJB2 as an inhibitor of HIV-1 infection, we isolated total CD4^+^ T cells from four people living with HIV (PLWH) whose viral loads were undetected (see Table S1 for their background data) and stimulated their CD4^+^ T cells transduced with lentiviral expression vectors (Fig. S5A). After the produced virus was washed out, the cells were divided into two groups for lentiviral expression of GJB2 or GFP in the presence of puromycin selection. The CD4^+^ T cells were washed with phosphate-buffered saline (PBS) at three time points to ensure that all drugs remaining in the cells were removed and would not continue to affect the spread of the produced HIV-1 virions. There was no obvious influence on CD4^+^ T cell proliferation among GJB2-expressing CD4^+^ T cells (Fig. S5B), and decreased viral titers were likely due to exogenous GJB2 expression (Fig. S5C and D). Therefore, GJB2 introduction inhibited HIV-1 spread in these CD4^+^ T cells *ex vivo*. Taken together, these *ex vivo* data validated that GJB2 inhibited HIV infection in CD4^+^ T cells.

### The anti-HIV-1 activity of GJB2 requires Ca^2+^

**Fig 4:**
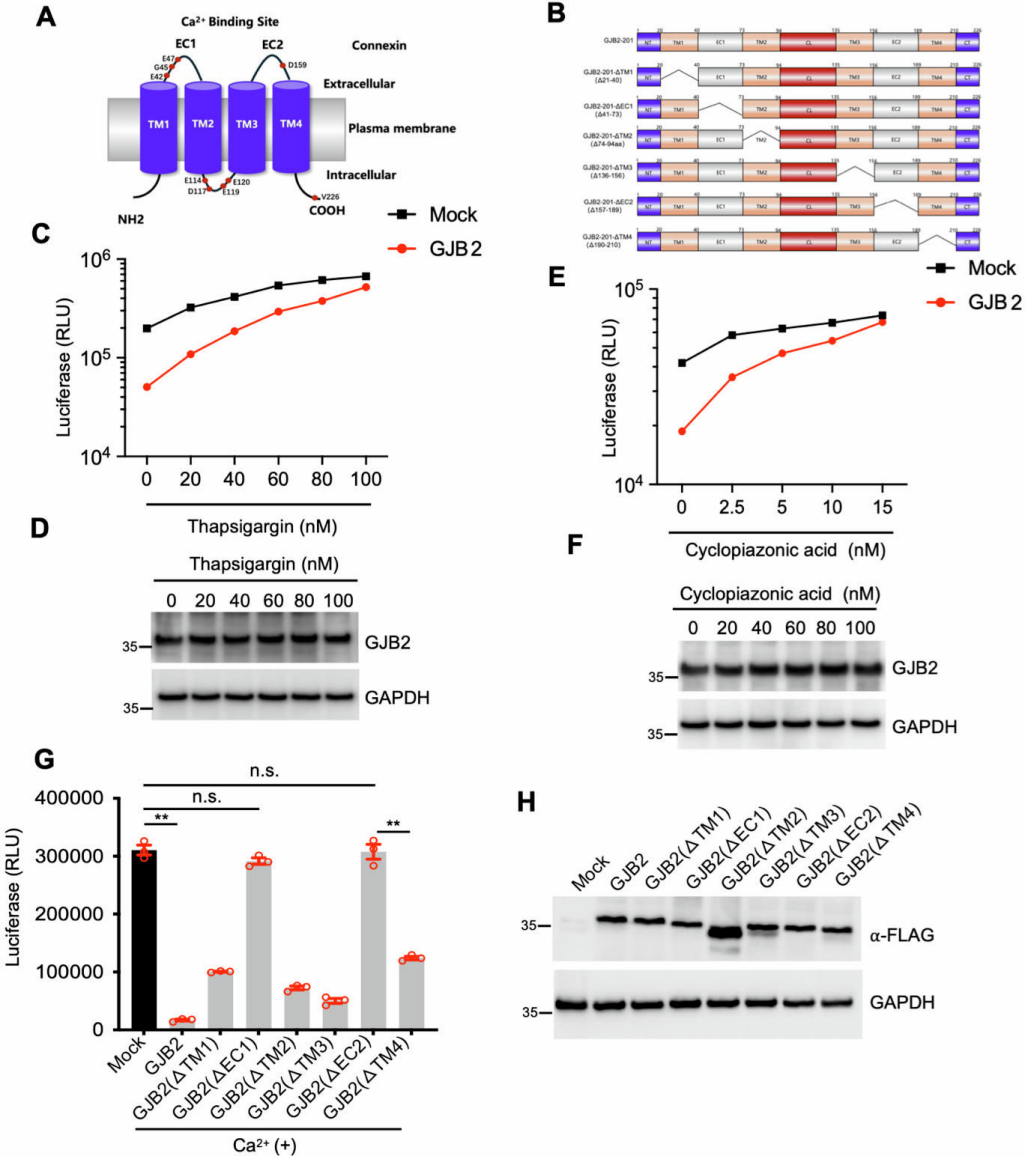
The anti-HIV-1 activity of GJB2 requires Ca^2+^. (**A**) GJB2 is located on the cell membrane and comprises 12 Cx proteins organized as hemichannels (two hexameric connexons). Cx comprises two extracellular loops, designated EC1 and EC2 (Ca^2+^-binding ectodomain), four transmembrane-spanning domains (TM1–TM4), one cytoplasmic loop (CL), one amino-(N) terminus (NT) region, and one carboxy-(C) terminus (CT) tail region. Each extracellular loop (EC1 and EC2) contains three conserved cysteine residues. (**B**) Schematic representation of the wild-type and mutant GJB2 domains. (C−F) 293T cells were transfected with a construct encoding non-tagged GJB2 or a mock expression construct. After 24 h of transfection, the cells were infected with 50 ng HIV-1_NL4-3.Luc.R−E−_ (VSV-G) in the presence or absence of thapsigargin (**C and D**) or cyclopiazonic acid (**E and F**) at the indicated doses. After 24 h of infection, the cells were lysed to measure luciferase reporter activity (**C, E**) and for western blotting to assess the levels of GJB2 and GAPDH using specific antibodies (**D, F**). Data are presented as the mean ± SD of three triplicates. (**G and H**) 293T cells were transfected with a construct encoding non-tagged wild-type GJB2 or truncated mutant or a mock expression construct as indicated. After 24 h of transfection, the cells were infected with 100 ng HIV-1_NL4-3.Luc.R−E−_ (VSV-G). The cells were lysed 24 h later to measure luciferase reporter activity (**G**) and for western blotting to assess the levels of GJB2 and GAPDH using specific antibodies (**H**). Data are presented as the mean ± SEM of three independent experiments. All western blotting data represent three independent experiments.

The GJB2 molecule comprises an N-terminal domain, four transmembrane domains (TM1–TM4), two extracellular loops (EC1 and EC2, interacting with Ca^2+^), a cytoplasmic loop, and a C-terminus (Fig. 4A and B). GJB2 and connexin hemichannels are sensitive to several intracellular and extracellular factors affecting various physiological processes and pathological states. One such factor is Ca^2+^ concentration, which ranges from 2 mM (extracellular) to 10 nM–100 nM (intracellular) and is key for regulating GJB2 conductance, making it a driver of multiple physiological properties (21, 28, 29). Therefore, we investigated the relationship between Ca^2+^ and GJB2. We evaluated the anti-HIV-1 activity of GJB2 in the presence or absence of thapsigargin or cyclopiazonic acid (a selective and membrane-permeable Ca^2+^ chelator), which depletes Ca^2+^ in the culture media. The inhibitory effect of GJB2 was substantially diminished in the presence of Ca^2+^ chelators at the concentrations used in the experiment (Fig. 4C through F), suggesting that GJB2 required Ca^2+^ to exert its anti-HIV-1 activity. GJB2 has two extracellular Ca^2+^-binding domains (Fig. 4A) to implement its function (30, 31). Accordingly, we generated mutants of GJB2, including ΔEC1 and ΔEC2 mutants that had lost the ability to bind to Ca^2+^ (Fig. 4B). We then examined the effect of wild-type and mutant GJB2 on HIV-1 infection, revealing that EC1 or EC2 deletion almost abrogated its antiviral activity (Fig. 4G and H). Additionally, all of the GJB2 transmembrane domains (TM1–TM4) appeared to be required for its anti-HIV-1 activity. This suggested that GJB2 was a multiple transmembrane protein and that disruption of its membrane configuration would influence its antiviral activity, which warrants future investigation. Overall, our findings demonstrated that the extracellular Ca^2+^-binding domains were critical for the anti-HIV-1 activity of GJB2 and that GJB2 required Ca^2+^ to inhibit HIV-1 infection in target cells.

### GJB2 inhibits HIV-1 virion attachment to the cell membrane

**Fig 5:**
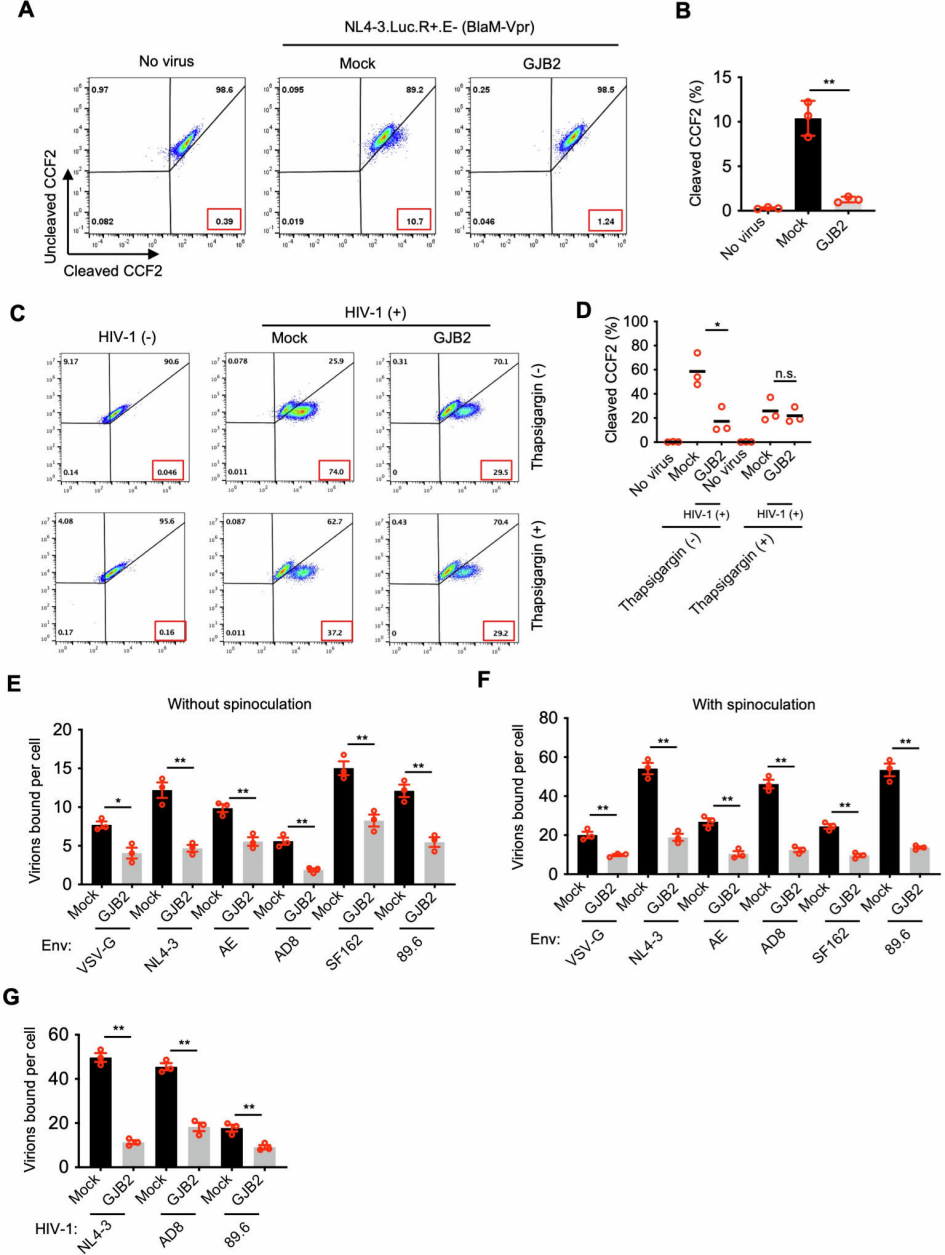
GJB2 inhibits HIV-1 virion attachment to the cell membrane. (**A and B**) BlaM-Vpr-based viral entry assay using VSV-G-pseudotyped HIV-1. The 293T cells were transfected with a construct encoding FLAG-tagged GJB2 or a mock expression construct. After 24 h, the cells were infected with 100 ng of VSV-G-pseudotyped HIV-1_NL4-3.Luc.R+E−_ (BlaM-Vpr) virions. Representative flow cytometry dot plots from three independent experiments are shown (**A**). The results of three independent experiments are presented as the mean ± SEM (**B**). **P < 0.01 (two-tailed unpaired Student’s *t*-test). (**C and D**) BlaM-Vpr-based viral entry assay using VSV-G-pseudotyped HIV-1. The 293T cells were transfected with a construct encoding FLAG-tagged GJB2 or a mock expression construct. After 24 h, the cells were infected with 100 ng of VSV-G-pseudotyped HIV-1_NL4-3.Luc.R+E−_ (BlaM-Vpr) virions in the presence or absence of thapsigargin (50 nM). Representative flow cytometry dot plots from three independent experiments are shown (**C**). The results of three independent experiments are presented as the mean ± SEM (**D**). *P < 0.05; n.s., not significant (two-tailed unpaired Student’s *t*-test). (**E and F**) HIV-1 attachment assay. CD4-, CXCR4-, and CCR5-expressing 293T cells were cotransfected with a construct encoding FLAG-tagged GJB2 or a mock expression construct. After 24 h, the cells were infected (**E**) or spinoculated (**F**) with 50 ng of HIV-1_NL4-3.Luc.R−E−_ (with different Env as indicated, CXCR4: NL4-3, CCR5: AE, AD8, and SF162, dual: 89.6) at 4°C for 2 h and washed with PBS. The cells were then lysed for p24 enzyme-linked immunosorbent assay (ELISA) to quantify the bound viral particles. The results of three independent experiments are presented as the mean ± SEM. *P < 0.05; **P < 0.01. (**G**) The CD4-, CXCR4-, and CCR5-expressing 293T cells were transfected with a construct encoding FLAG-tagged GJB2 or a mock expression construct. After 24 h, the cells were spinoculated with 50 ng of the indicated HIV-1 isolate at 4°C for 2 h and washed with PBS. The cells were then lysed for p24 ELISA to quantify the bound viral particles. The results of three independent experiments are presented as the mean ± SEM (**B**). *P < 0.05; **P < 0.01 (two-tailed unpaired Student’s *t*-test).

To elucidate the underlying molecular mechanism of GJB2 inhibition of HIV-1 infection, we analyzed the entry efficiency of HIV-1 particles into target cells in the presence of GJB2 using a β-lactamase (BlaM)-fused Vpr entry assay (32). This assay assessed the entry of virions carrying the BlaM-Vpr fusion protein by measuring the activity of the BlaM delivered to the target cells. HIV-1_NL4-3.Luc.R+E−_ viruses (VSV-G) carrying the BlaM-Vpr reporter protein were used to infect 293T cells in the presence or absence of GJB2. The entry efficiency of HIV-1 was markedly reduced in the presence of GJB2 compared with that of the control (Fig. 5A), and this result was observed in three independent experiments (Fig. 5B). Thus, GJB2 substantially impaired the entry of VSV-G-pseudotyped HIV-1 virions into target cells. In addition, we found that the GJB2-mediated inhibition of viral entry requires presence of Ca^2+^ (Fig. 5C and D). Next, we used HIV-1 virion attachment assays to investigate whether GJB2 restricted HIV-1 virion attachment to the target cells, revealing that GJB2 significantly impaired HIV-1 attachment (Fig. 5E and F). Consistently, GJB2 significantly impaired the attachment of CXCR4-, CCR5-, and dual-tropic HIV-1 isolates to the target cells (Fig. 5G). Therefore, GJB2 may interfere with HIV-1 virion attachment to the cell membrane, restricting HIV-1 infection of target cells.

### GJB2 inhibits HIV-1 spread in macrophages and DCs

GJB2 is highly expressed in myeloid lineages. Thus, we explored the anti-HIV activity of GJB2 in primary macrophages. Monocytes were isolated from the peripheral blood mononuclear cells (PBMCs), and monocyte-derived macrophages (MDMs) were generated by stimulating monocytes with human granulocyte-macrophage colony-stimulating factor (GM-CSF) and human M-CSF.

**Fig 6:**
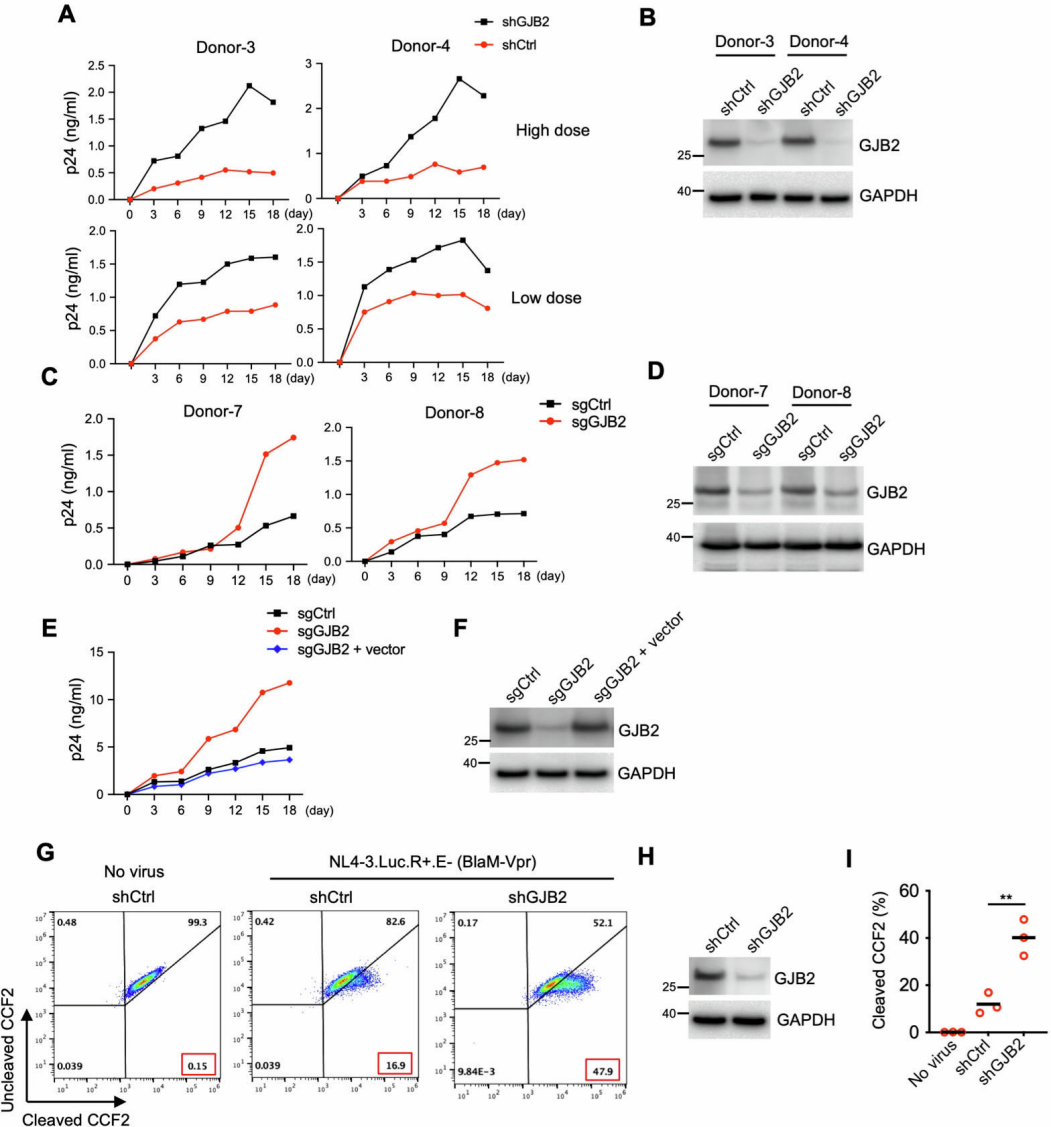
GJB2 inhibits HIV-1 spread in macrophages. (**A and B**) Lentiviral shRNA-transduced MDMs were infected with 5 ng (low dose) or 50 ng (high dose) of HIV-1_AD8_ for 18 days. Viral production was measured using p24 enzyme-linked immunosorbent assay (ELISA) at the indicated time points (**A**). The aliquoted cells were lysed for western blotting to assess the GJB2 and GAPDH levels (**B**). (**C and D**) Lentiviral CRISPR-Cas9-transduced MDMs (with single-guide RNAs [sgRNAs] to disrupt *GJB2* loci) were infected with 25 ng of HIV-1_AD8_ for 18 days. Viral production was measured using p24 ELISA at the indicated time points (**C**). The aliquoted cells were lysed for western blotting to assess the GJB2 and GAPDH levels (**D**). (**E and F**) Lentiviral CRISPR-Cas9-transduced MDMs (with sgRNAs to disrupt *GJB2* loci) were transduced with or without a lentiviral expression vector encoding non-tagged GJB2 and then infected with 50 ng HIV-1_AD8_ for 18 days. Viral production was measured using p24 ELISA at the indicated time points (**E**). The MDMs were lysed for western blotting to assess GJB2 and GAPDH levels (**F**). (**G−I**) The BlaM-Vpr-based viral entry assay using a VSV-G-pseudotyped HIV-1. Lentiviral shRNA-transduced MDMs were infected with 100 ng of VSV-G-pseudotyped HIV-1_NL4-3.Luc.R+E−_ (BlaM-Vpr) virions. The cleavage of intracellular CCF2 was measured using flow cytometry (**G**). The aliquoted cells were lysed for western blotting to assess the GJB2 and GAPDH levels (**H**). The results of three independent experiments are presented as the mean ± SEM (**I**). **P < 0.01 (two-tailed unpaired Student’s *t*-test).

First, we depleted endogenously expressed GJB2 using a lentiviral short hairpin RNA (shRNA) vector targeting GJB2, and then we infected these cells with CCR5-tropic replication-competent HIV-1_AD8_ (Fig. 6A and B). We found that the GJB2 silencing noticeably enhanced the spread of HIV-1 during the 18 day infection period in MDMs. Additionally, a similar enhancement of HIV-1 spread in MDMs was observed when we used the CRISPR-Cas9 technique to disrupt *GJB2* loci (Fig. 6C and D). As expected, viral inhibition was rescued in these *GJB2* loci-disrupted cells by supplementing an exogenous non-tagged GJB2 lentiviral vector (Fig. 6E and F).

Moreover, as shown in Fig. S6A andB, silencing GJB2 also promoted cell-to-cell HIV-1 transmission. Notably, the percentage of Gag-positive cells increased from 1.81% to 10.1% in the absence of GJB2. In contrast, when a transwell culture was used to block cell-to-cell contact, the percentage of Gag-positive cells increased only from 1.31% to 4.63%. Overall, these data indicated that endogenous GJB2 inhibited HIV-1 transmission in primary macrophages.

We also investigated the anti-HIV activity of GJB2 in monocyte-derived dendritic cells (MDDCs). The shRNA-mediated depletion of GJB2 enhanced the spread of HIV-1 over the 18 day infection period in MDDCs, which became more permissible to the virus following pretreatment with virus-like particle (VLP)-Vpx to deplete SAMHD1 protein (Fig. S7A and B). We obtained similar results when the *GJB2* loci were disrupted using the CRISPR-Cas9 technique (Fig. S7C and D). Collectively, these data indicated that GJB2 inhibited HIV-1 transmission in both macrophages and DCs.

We next investigated whether GJB2 adopted the same molecular mechanism to inhibit HIV-1 infection by impairing virion attachment to primary macrophages. The BlaM-fused Vpr entry assays demonstrated that GJB2 silencing consistently increased HIV-1 entry into MDMs (Fig. 6G and H). Moreover, GJB2 depletion significantly promoted the fusion of HIV-1 virions to macrophages in three independent experiments (Fig. 6I). Taken together, these findings indicated that GJB2 also impaired HIV-1 virion fusion to macrophages to inhibit viral transmission.

### IL-4 enhances HIV-1 infection in macrophages by downregulating GJB2 expression

**Fig 7:**
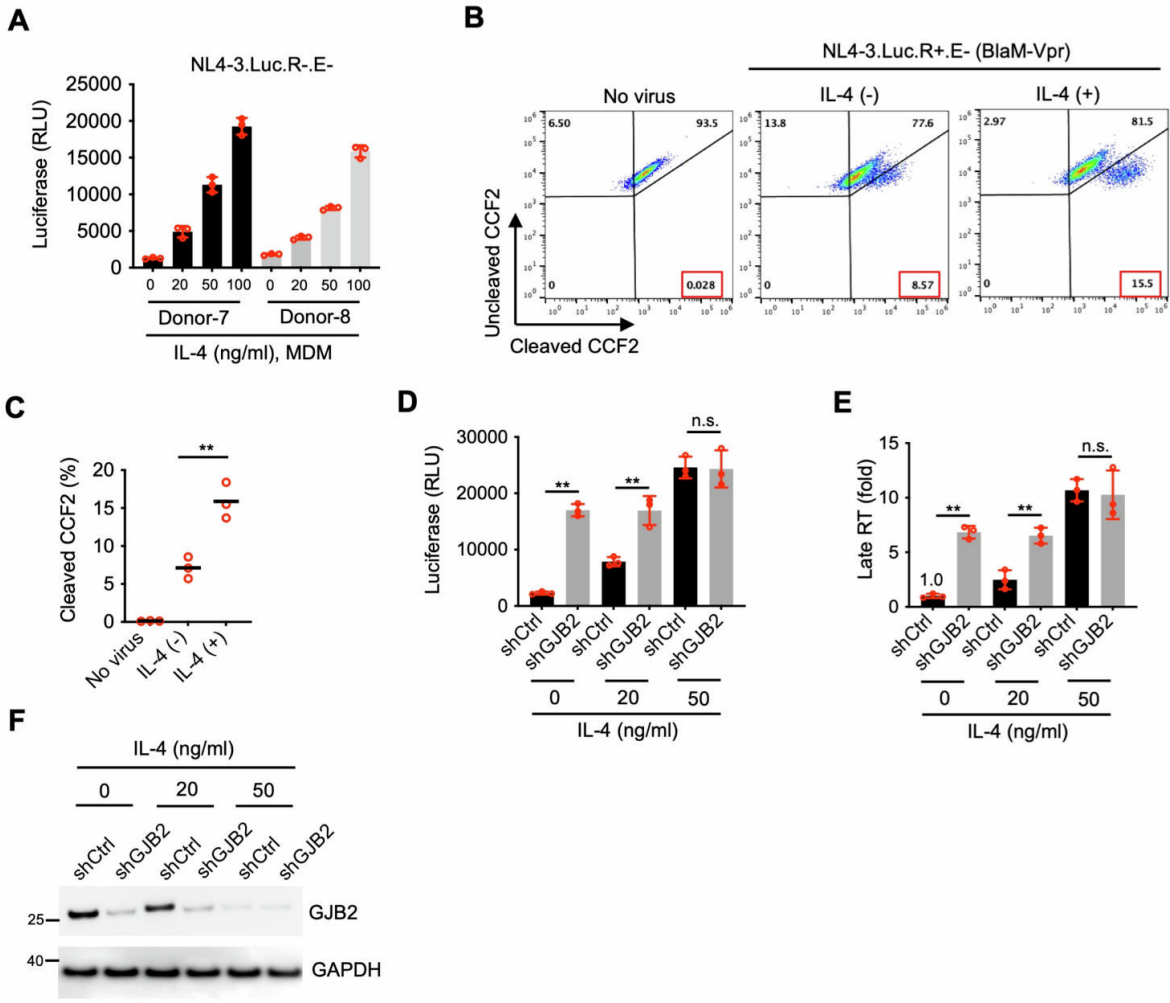
IL-4 enhances HIV-1 infection in macrophages by downregulating GJB2 expression. (**A**) MDMs were infected with 10 ng of HIV-1_NL4-3.Luc.R−E−_ (VSV-G) in the presence or absence of IL-4 (50 ng/mL). The cells were lysed 24 h after infection to measure luciferase reporter activity. Data are presented as the mean ± SD from three triplicates. (**B and C**) BlaM-Vpr-based viral entry assay using VSV-G-pseudotyped HIV-1. MDMs were infected with 200 ng of VSV-G-pseudotyped HIV-1_NL4-3.Luc.R+E−_ (BlaM-Vpr) virions in the presence or absence of IL-4 (50 ng/mL). The cleavage of intracellular CCF2 was measured using flow cytometry (**B**). Data from three independent experiments are presented as the mean ± SEM (**C**). (**D−F**) Lentiviral shRNA-transduced MDMs were infected with 100 ng of HIV-1_NL4-3.Luc.R−E−_ (VSV-G) for 24 h. Then the cells were lysed to measure luciferase reporter activity (**D**) and for western blotting to assess GJB2 and GAPDH expression (**F**). Total DNA was extracted for qPCR to measure viral late RT (**E**). Data are presented as the mean ± SEM from three independent experiments. **P < 0.01; n.s., not significant (two-tailed unpaired Student’s *t*-test). All western blotting data represent three independent experiments.

IL-4 is a well-known promoter of HIV-1 infection in primary macrophages (18–20) and reduces GJB2 expression in macrophages (Fig. 2D and E). Therefore, we investigated whether GJB2 downregulation enhanced HIV-1 infection. First, we experimentally confirmed that IL-4 stimulated HIV-1 infection in primary macrophages (Fig. 7A). Then, we investigated whether IL-4 enhanced HIV-1 entry in macrophages. Results from three independent, healthy donors demonstrated that IL-4 enhanced HIV-1 entry into macrophages (Fig. 7B and C). To investigate whether this reduction in GJB2 enhanced infection, we first silenced GJB2 in MDMs using RNA interference in the presence or absence of IL-4 treatment (20 or 50 ng/mL). In the absence of IL-4, GJB2 knockdown still promoted HIV-1 infection in macrophages (shCtrl vs shGJB2 without IL-4) (Fig. 7D). In the shRNA nontarget control (shCtrl)-transduced macrophages, IL-4 consistently enhanced HIV-1 infection and reduced GJB2 expression in a dose-dependent manner (Fig. 7D through F). In contrast, GJB2 silencing diminished the IL-4-mediated enhancement (shCtrl vs shGJB2 with IL-4). In the absence of GJB2 (Fig. 7D, in shGJB2), IL-4 (20 or 50 ng/mL) did not enhance HIV-1 infection. This shRNA-mediated depletion of GJB2 almost abrogated the IL-4-mediated enhancement at 50 ng/mL (shCtrl vs shGJB2 with IL-4 at 50 ng/mL). The GJB2 protein level (Fig. 7F) was also inversely related to the reporter luciferase activity and late RT levels (Fig. 7D and E). When GJB2 was silenced, IL-4 reduced the level of GJB2 protein to a greater extent at 50 ng/mL than at 20 ng/mL. Therefore, reduced GJB2 levels inversely enhanced HIV-1 replication to some extent (shGJB2 with 0 ng/mL IL-4 vs shGJB2 with 50 ng/mL IL-4). Collectively, these findings indicated that GJB2 depletion using shRNA impaired the IL-4-mediated enhancement of HIV-1 infection.

**Fig 8:**
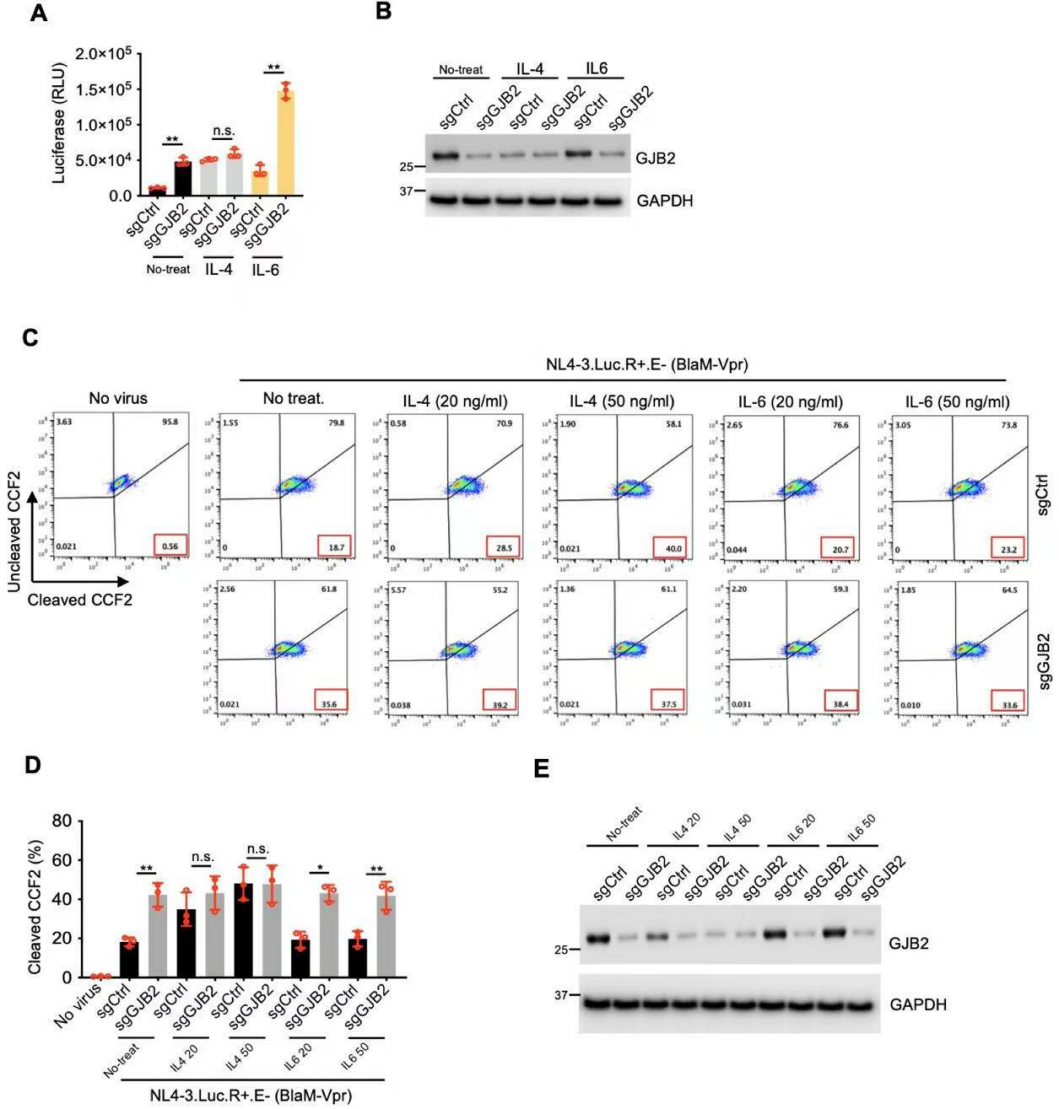
IL-4, but not IL-6, downregulates GJB2 to enhance HIV-1 infection in macrophages. (**A and B**) Lentiviral CRISPR-Cas9-transduced MDMs (with single-guide RNAs [sgRNAs] to disrupt *GJB2* loci) were infected with 100 ng of HIV-1_NL4-3.Luc.R−E−_ (VSV-G) in the presence or absence of IL-4 (50 ng/mL) or IL-6 (50 ng/mL). After 24 h, the cells were lysed to measure luciferase reporter activity (**A**) and for western blotting to assess GJB2 and GAPDH expression (**B**). Data are presented as the mean ± SEM from three independent experiments. (**C–E**) Lentiviral CRISPR-Cas9-transduced MDMs (with sgRNAs to disrupt *GJB2* loci) were infected with 100 ng VSV-G-pseudotyped HIV-1_NL4-3.Luc.R+E−_ (BlaM-Vpr) virions with or without IL-4 or IL-6 at the indicated doses. The cleavage of intracellular CCF2 was measured using flow cytometry (**C**). The results of three independent experiments are presented as the mean ± SEM (**D**). Cells were lysed for western blotting to assess GJB2 and GAPDH expression (**E**). *P < 0.05 and **P < 0.01; n.s., not significant (two-tailed unpaired Student’s *t*-test). All western blotting data represent three independent experiments.

To confirm our findings, we used the CRISPR-Cas9 technique to disrupt *GJB2* loci in primary macrophages in the presence or absence of IL-4 or IL-6 and then infected these cells with a VSV-G-pseudotyped HIV-1_NL4-3.Luc.R−E−_ reporter virus. Our results showed that both IL-4 and IL-6 promoted HIV-1 infection without disrupting the *GJB2* loci (Fig. 8A and B). However, when the *GJB2* loci were disrupted, IL-4-mediated enhancement was impaired (sgCtrl vs sgGJB2), but IL-6-mediated enhancement was unaffected (sgCtrl vs sgGJB2). Therefore, IL-4 but not IL-6 downregulated GJB2 to promote HIV-1 infection in macrophages.

We also used BlaM-fused Vpr entry assays to investigate whether the IL-4-mediated enhancement of HIV-1 entry in macrophages was abolished in the absence of GJB2. The results showed that HIV-1 entry was enhanced in the presence of IL-4 but not in the presence of IL-6 (Fig. 8C and D), and the disruption of *GJB2* loci by CRISPR-Cas9 (Fig. 8E) abrogated the IL-4-enhanced but not IL-6-enhanced HIV-1 entry into primary macrophages (Fig. 8D). This indicates that IL-4 promotes HIV-1 entry by downregulating GJB2, suggesting that the underlying molecular mechanism of IL-4-enhanced entry is distinct from that of IL-6. In contrast, IL-6 does not affect viral entry. Instead, it may enhance HIV-1 infection by promoting reverse transcription or other downstream events following viral entry. To summarize, we identified GJB2 as a new antiviral factor whose expression was reduced by IL-4, thereby enhancing HIV-1 entry into macrophages.

## DISCUSSION

The loss of T cells with helper function is a central feature of HIV-1 infection. However, other signs of immune dysregulation, such as early loss of cellular responses to recall antigens, polyclonal B cell activation, and hypergammaglobulinemia, are present in all PLWH and are attributed to Th2-produced cytokines, such as IL-4 and IL-13 (33, 34). IL-4 is a pleiotropic cytokine produced by Th2 cells (a subpopulation of CD4^+^ T cells), basophils, and mast cells. Individuals with HIV-1 infection show increased production of IL-4, the primary Th2-defining cytokine, as evidenced by an increased frequency of T cell clones with the Th2 phenotype and intracellular staining of IL-4 (35–38). Furthermore, higher levels of virus replication were reported as correlated with increased IL-4 levels in lymph nodes of children infected with HIV-1 (39). Notably, IL-4 has also been reported to enhance HIV-1 infection in primary monocytes/macrophages (18–20).

In our study, we identified GJB2 as a new antiviral factor in myeloid cells. GJB2 is mainly and constitutively expressed in macrophages and DCs but not in CD4^+^ lymphocytes and Jurkat, 293T, and HeLa cells. We found that *GJB2* silencing or knockout enhanced HIV-1 spread in both macrophages and DCs, suggesting GJB2 as a new antiviral factor in myeloid cells. Although IFNs did not upregulate GJB2 expression in either CD4^+^ T cells or primary macrophages, we discovered that IL-4 downregulated GJB2 expression in macrophages. This finding prompted us to investigate whether IL-4-induced downregulation enhanced HIV-1 infection. Our findings showed that GJB2 silencing impaired IL-4- but not IL-6-mediated enhancement of HIV-1 infection, suggesting that IL-4-mediated downregulation of GJB2 expression enhanced HIV-1 infection. Additionally, unlike IL-4, the IL-6-mediated enhancement of HIV-1 infection in macrophages was unrelated to GJB2. Moreover, we confirmed that the IL-4-enhanced HIV-1 entry into macrophages was impaired in the absence of GJB2.

We investigated the underlying molecular mechanism of GJB2 inhibition of HIV-1 infection, revealing that GJB2 interfered with HIV-1 virion attachment to target cells, thereby inhibiting HIV-1 infection. Furthermore, the viral-encoded accessory proteins did not affect the antiviral activity of GJB2. Moreover, the inhibitory effect of GJB2 was independent of HIV-1 glycoproteins. Although HIV-1 infection with cell-free viruses has been largely documented, the dominant mode of infection is probably cell-to-cell transmission (40, 41). Upon investigating this possibility, we discovered that GJB2 inhibited cell-to-cell HIV-1 transmission. However, further study is necessary to confirm whether GJB2 interferes with HIV-1 dissemination by cell-to-cell transfer in myeloid cell targets. Importantly, when we reintroduced GJB2 into CD4^+^ T cells isolated from anti-retroviral therapy (ART)-treated patients, we observed that GJB2 overexpression still inhibited HIV-1 spread in these cells *ex vivo*, suggesting a potential application for combating HIV/AIDS.

GJB2 exhibited a broad antiviral activity against retroviruses other than HIV-1, such as HIV-2, SIV, EIAV, and MLV. Thus, future investigations are necessary to elucidate whether GJB2 also inhibits the attachment of these virions to host cells. Using membrane-permeable Ca^2+^ chelators, we discovered that GJB2 required Ca^2+^ to exert its antiviral activity. We confirmed this result by demonstrating the loss of antiviral activity in GJB2 mutants without the extracellular Ca^2+^-binding domains EC1 or EC2. Interestingly, disruption of the TM1, TM2, TM3, or TM4 domains impaired GJB2’s antiviral activity, suggesting that these domains are critical for its correct configuration on the cell membrane. Overall, our study provides insights into the ability of myeloid lineage cells to interfere with HIV-1 infection through GJB2 and partially answers the question of how IL-4 enhances infection by downregulating GJB2. Further elucidation of the interaction between GJB2 and other viruses may help develop therapeutic strategies targeting viral infections.

## MATERIALS AND METHODS

### People living with HIV-1

The people living with HIV-1 on ART with viral loads of <50 copies/mL and untreated PLWH (Table S1) were enrolled in this study. PBMCs from PLWH were prepared using Ficoll-Hypaque density gradient centrifugation. CD4^+^ T cells were isolated from PBMC by negative selection with human CD4^+^ T cells enrichment cocktail (StemCell Technologies).

### Cells and cell culture reagents

The mouse cell line NIH3T3 and human cell lines 293T and TZM-bl were cultured in Dulbecco Modified Eagle Medium (Gibco), and the human cell lines THP-1 and Jurkat were cultured in RPMI 1640 medium (Gibco). Both media were supplemented with 10% fetal bovine serum (FBS; Gibco), 100 U/mL penicillin, and 100 mg/mL streptomycin. Plasmids were transfected into the 293T cells using Lipofectamine 2000 (Invitrogen). PBMCs obtained from healthy blood donors were purified using Ficoll-Hypaque density gradient centrifugation. CD4^+^ T cells or monocytes were isolated from the PBMCs using negative selection with human CD4^+^ T cells or a CD14-positive enrichment cocktail (StemCell Technologies). The CD4^+^ T cells were stimulated by adding CD3/CD28 activator magnetic beads (Invitrogen) and IL-2 (50 U/mL; Biomol) to the culture medium for 2 days. The isolation and culture of monocytes, MDMs, and MDDCs were performed as previously described (42, 43). MDMs were generated by stimulating monocytes with 10 ng/mL recombinant human GM-CSF (R&D Systems) and 50 ng/mL recombinant human M-CSF (R&D Systems) for 7 days. MDDCs were generated by incubating CD14-purified monocytes in Iscove Modified Dulbecco Medium (Gibco) supplemented with 10% FBS, 2 mM L-glutamine, 100 IU/mL penicillin, 100 mg/mL streptomycin, 10 mM HEPES, 1% nonessential amino acids, 1 mM sodium pyruvate, 10 ng/mL GM-CSF, and 50 ng/mL IL-4 (Miltenyi Biotec). On day 4, two-thirds of the culture medium was replaced with fresh medium containing GM-CSF and IL-4, and immature MDDCs were harvested on day 6 for use in our experiments. Lipofectamine 3000 (Thermo Fisher Scientific) was used to transfect small interfering RNA into MDMs or MDDCs.

### Plasmids

GJB2 expression vectors (OriGene) or their open reading frames (ORFs) underwent *de novo* cloning into vector pCMV-3Tag-2A (Addgene). HIV-1 reporter vector NL4-3.Luc.R−E− and proviral vectors NL4-3, 89.6, SIV_mac239_, SIV_agm_, HIV-2_Rod_, and MLV were obtained from the NIH AIDS Program. The proviral vector HIV-1_AD8_ was gifted by Dr. E. Freed. Reporter vectors SIV_mac239_-luc, HIV-2_Rod_-luc, and MLV-luc were gifted by Dr. G. Gao (44). Vectors to construct Vpx (−)/(+) VLP were gifted by Dr. Nathaniel Landau (45). The experiments related to wild-type HIV-1 virions were performed in Biological Safety Level 3 (BSL-3) facility.

### RNA interference in THP-1 cells, MDMs, MDDCs, and CD4^+^ T cells

A microRNA-adapted shRNA lentivirus was introduced into MDMs and MDDCs, as previously described (42, 43), for shRNA-mediated silencing of *GJB2* (RHS4430-200259317: 5′-TAGCAAATAACACAATTCA-3′ targeting ORF; RHS4430-200160676: 5′-AAGACAGGCATAGAATTAG-3′ targeting 3′-untranslated region (UTR)or as a nontargeting control (cat #: RHS4346). Briefly, freshly isolated monocytes were treated with VLP-Vpx and transduced with shRNA lentivirus particles. Following puromycin selection, the cells were infected with replication-competent CCR5-tropic HIV-1 for 6 h and washed twice with cold PBS to remove the input virus. Recombinant lentiviruses for CRISPR (single-guide RNAs [sgRNAs] and Cas9) were generated via the transient transfection of vectors (GenScript) and packaging plasmids in 293T cells, and the target cells were infected with a lentivirus expressing Cas9 and a lentivirus expressing sgRNAs (sgGJB2-1: 5′-GCGGTTTGCTCTGCGTCGGG and sgGJB2-2: 5′-TCGCATTATGATCCTCGTTG), followed by puromycin and blasticidin double resistance selection. Plasmids for CRISPR, which transiently express sgRNA and Cas9 in mammalian cells, were purchased from GenScript.

### Enzyme-linked immunosorbent assay (ELISA) for HIV virion detection

The levels of p24 in the culture supernatants were measured via ELISA per the manufacturer’s instructions (HIV-1 p24 antigen capture assay, ABL Corporation).

### Quantification of late RT products of HIV-1

HIV-1 producer 293T cells were washed twice with PBS to remove the transfected HIV-1 plasmid before infection. The obtained viral stocks were treated with DNase I (Takara Bio) for 1 h at 37°C, and the DNA was extracted using a DNeasy Blood & Tissue Kit (QIAGEN). Real-time polymerase chain reaction (qPCR) was performed to quantify viral late RT with primers Late RT forward (5′-AGCAGGAACTACTAGTACCC-3′) and Late RT reverse (5′- TTGTCTTATGTCCAGAATGC-3′) as described previously (46).

### Luciferase detection assay

Luciferase activity in the cell lysates was quantified as relative luminescence units per the manufacturer’s instructions (Promega).

### Isolation of membrane-associated proteins

Membrane-associated proteins were isolated using a Mem-PER Plus Membrane Protein Extraction Kit (Invitrogen) (42). Briefly, cells were harvested from a culture suspension by centrifugation (300 × *g*, 5 min). The resultant cell pellet was washed with 3 mL Cell Wash Solution and centrifuged (300 × *g*, 5 min), and the supernatant was discarded. Then the cell pellet was resuspended in 1.5 mL Cell Wash Solution, transferred to a new tube, and centrifuged (300 × *g*, 5 min). After discarding the supernatant, the cell pellet was mixed with 0.75 mL permeabilization buffer to obtain a homogeneous cell suspension and incubated for 10 min at 4°C. The permeabilized cells were centrifuged (16,000 × *g*, 15 min), and the resultant supernatant containing cytosolic proteins was removed and transferred to a new tube for detection. The pellet was resuspended in 0.5 mL solubilization buffer and incubated with constant mixing for 30 min at 4°C. Then the mixture was centrifuged (16,000 × *g*, 4°C, 15 min), and the supernatant containing the solubilized membrane and membrane-associated proteins was transferred to a new tube for western blot analysis.

### HIV-1 attachment assay

We performed an HIV-1 attachment assay as previously described (34, 35). Briefly, 3 × 10^5^ TZM-bl cells or 2 × 10^5^ stimulated CD4^+^ T cells were spinoculated with HIV-1 (1,200 × *g*, 25°C, 2 h). Then the infected cells were washed five times with cold medium to remove the unbound viral particles and lysed with 0.5% Triton X-100 for quantification of cell-associated p24 Gag protein using ELISA. Virion equivalents were determined assuming an average of 1,500 p24 Gag molecules per HIV-1 particle, in other words, 15,800 viral particles per pg of p24 Gag.

### BlaM-Vpr-based viral entry assay

HIV-1 particles incorporating a fusion protein comprising Vpr and BlaM reporter protein were produced by cotransfection of NL4-3.Luc.R+E− with an expression vector encoding BlaM-Vpr. After quantification by p24 ELISA, 100 ng of the p24 virus was incubated with target cells at 37°C for 4 h to allow viral entry. After washing three times with Hank’s balanced salt solution (HBSS; Thermo Fisher Scientific), the cells were resuspended and loaded with 1 µM CCF2-AM dye (Thermo Fisher Scientific), a fluorescent substrate for BlaM, in HBSS containing 1 mg/mL Pluronic F-127 surfactant (Thermo Fisher Scientific) and 0.001% acetic acid for 1 h at room temperature, and then washed twice with HBSS. The BlaM reaction (cleavage of the intracellular CCF2 dye by BlaM-Vpr) was developed for 14 h at room temperature in HBSS supplemented with 10% FBS. Then the cells were washed three times with PBS and fixed in 1.2% paraformaldehyde. Fluorescence was monitored at 520 and 447 nm using an ID7000 flow cytometer (Sony).

### Western blotting and the antibodies

The isolated membrane-associated proteins underwent sodium dodecyl sulfate-polyacrylamide gel electrophoresis, and then the separated proteins were placed onto a polyvinylidene fluoride membrane for western blotting using a standard method. The following antibodies were used: polyclonal Rabbit anti-GJB2 (cat #: 71-0500, 1:1,000; Thermo Fisher), rabbit anti-GAPDH (cat #: PA1-987, 1:1,000; Thermo Fisher Scientific), mouse monoclonal anti-FLAG M2 antibody (cat #: F1804, 1:5,000; Sigma-Aldrich), and mouse Ig-HRP (cat #: ab6789, 1:1,000; Abcam).

### Microscopy

MDMs were treated with a human Fc receptor blocking solution (BioLegend), before staining with the antibody. The cells were viewed and images were taken using an LSM 980 microscope with an Airyscan 2 Imaging System (Zeiss).

### Real-time polymerase chain reaction

Total RNA was extracted from the cells using TRIzol reagent (Invitrogen), per the manufacturer’s instructions, and dissolved in 100 µL of diethyl pyrocarbonate-treated water. Then 1 µg of the purified RNA was treated with DNase I, Amplification Grade (Invitrogen) for 10–15 min at room temperature, according to the manufacturer’s instructions. The RNA was immediately primed with oligo-dT and reverse-transcribed using Superscript III Reverse Transcriptase (Invitrogen). qPCR analysis was performed using the ΔΔCT method. GAPDH was used as the internal control for normalization of the results. The primer pairs and sequences are detailed in Table S2.

### Statistical analysis

Statistical analysis was performed using Prism v.6.0 (GraphPad Software). Unless otherwise stated, a two-tailed unpaired Student’s *t*-test was used for statistical comparison between groups. Each experiment was independently performed three times. Data were presented as the mean ± standard error of the mean or mean ± standard deviation.

## Data Availability

All study data are included in the article or the Supplementary Information Appendix.

## SUPPLEMENTAL MATERIAL

Supplemental file mbio.01626-25-s0001.pdf Supplemental figures and tables.

ASM does not own the copyrights to Supplemental Material that may be linked to, or accessed through, an article. The authors have granted ASM a non-exclusive, world-wide license to publish the Supplemental Material files. Please contact the corresponding author directly for reuse.
