# Supplementary material for: IL-4 downregulates gap junction protein connexin 26 to promote HIV-1 infection in macrophages
Source: mBio. 2025 Sep 22;16(11):e01626-25. doi: 10.1128/mbio.01626-25 (PMC12607889; doi:10.1128/mbio.01626-25)
Supplement: Supplemental file — Supplemental figures and tables. [file mbio.01626-25-s0001.pdf]

Figure S1. HIV-1 accessory proteins do not affect the inhibitory effect of GJB2.

(A and B) 293T cells were cotransfected with a construct encoding GFP-tagged GJB2 or a mock expression construct in the presence of FLAG-tagged Nef, Vif, Vpr, Vpu, Vpx or a negative control expression construct as indicated. After 24 h of cotransfection, the cells were infected with 50 ng HIV-$1_{NL4-3.Luc.R-.E-}$ (VSV-G). After 24 h of infection, the cells were lysed to measure luciferase reporter activity (A) and for western blotting to assess the levels of exogenous proteins and GAPDH using specific antibodies (B). Data are presented as the mean ± SD of three triplicates. **$p < 0.01$; *$p < 0.05$; NS, not significant (two-tailed, unpaired Student's *t*-test). Western blotting results are representative of three independent experiments. (C and D) The band intensities of GJB2, Nef, Vif, Vpr, Vpu, and Vpx were measured and normalized against GAPDH using ImageJ software, based on data from three independent experiments.

Figure S2. IFNs did not induce GJB2 expression.

(A and B) Stimulated $CD4^+$ T cells (A) or MDMs (B) were treated with or without IFN-α, -β, or -γ for 24 or 48 h (1000 U/mL). Total RNA was extracted for qPCR to measure the transcript levels of *GJB2*, *ISG15*, and *CXCL10* normalized against the *GAPDH* levels. Data are presented as the mean ± SEM from three independent experiments. **$P < 0.01$; ns, not significant (two-tailed unpaired Student *t*-test).

Figure S3. GJB2 is localized on the cell membrane.

(A) 293T cells were transfected with a construct encoding non-tagged, C-terminus FLAG-, or GFP-tagged GJB2 or a mock expression construct as indicated. After 24 h of

transfection, the cells were infected with 50 ng HIV-$1_{NL4\text{-}3.Luc.R-E-}$ (VSV-G). The cells were lysed after 24 h to measure luciferase reporter activity, and western blotting was performed to assess GJB2 and GAPDH levels. Data are presented as the mean ± SEM from three independent experiments. **P < 0.01 (two-tailed unpaired Student *t*-test). The band intensities of western bloting were measured and normalized against GAPDH using ImageJ software (lower panle), based on data from three independent experiments. (B) 293T cells or (C) stimulated $CD4^+$ T cells were transfected or electroporated with constructs encoding GFP-tagged or non-tagged GJB2 or a mock. After 24 h of transfection, viable cells were isolated and stained with rabbit anti-GJB2 or isotype (IgG) antibodies to measure the levels of GJB2 on the cell surface using PE-conjugated goat anti-rabbit antibodies. (D) 293T cells or (E) stimulated $CD4^+$ T cells expressing a C-terminus GFP-tagged GJB2 were visualized using microscopy. The cell nuclei were stained with DAPI. Scale bars: 10 μm. (F) MDMs were fixed and immunostained with anti-GJB2 or anti-Na^+/K^+ ATPase antibodies, or non-specific rabbit or mouse IgG and, which were probed with a second antibody conjugated with Alexa Fluor 488 or 647. Cell nuclei were stained with DAPI. Scale bars, 10 μm. (G) MDMs or MDDCs were lysed, and membrane-bound and cytosolic proteins were separated to assess the levels of GJB2, Na^+/K^+-ATPase, and GAPDH using western blotting with specific antibodies. (H) The band intensities in (G) were measured and normalized against β-actin using ImageJ software, based on data from three independent experiments. All blotting and flow cytometry data are representative of three independent experiments.

Figure S4. GJB2 inhibits cell-to-cell HIV-1 transmission.

(A) An equal number of stimulated CD4^{+} T cells, transduced with lentiviral expression vectors encoding GJB2 or a mock control, were stained with CellTrace Violet. For cell-to-cell infection assays, the transduced CD4^{+} T cells served as target cells, and ACH-2 cells were used as donor cells at a 2:1 target-to-donor ratio. Prior to co-culture, ACH-2 cells were stimulated with either 50 ng/mL PMA or DMSO for 24 h. Target and donor cells were then co-cultured either directly or separated using transwells with a 0.4-μm pore size. In the transwell setup, virus-producing ACH-2 cells were placed in the upper chamber and CD4^{+} T cells in the lower chamber. At 48h after co-culture, Gag-positive cells were analyzed by flow cytometry. (B) The results of five independent experiments are presented as the mean ± SEM (D). *P < 0.05 and **P < 0.01 (two-tailed unpaired Student *t*-test).

Figure S5. GJB2 inhibits HIV-1 spread in CD4^{+} T cells of PLWH *ex vivo*.

(A–D) Schematic representation of the experimental design for PLWH (A). CD4 $^{+}$T cells isolated from four PLWH (see Supplementary Table 1 for their background data) were stimulated with CD3CD28 activators and IL-2 (100 U/mL) for 3 days. The cells were washed, aliquoted, and transduced with lentiviral expression vectors of GJB2 or GFP. After 2 days of transduction, they were washed, and puromycin (0.5 ng/mL) was added to the culture media in the presence of stimulation (CD3CD28 activators and IL-2). After 2 days of puromycin selection, the cells were washed to remove the produced virions. The same number of cells (GJB2 or mock) were resuspended in culture media in the presence of stimulation (CD3CD28 activators and IL-2), and cell proliferation was assessed by CellTrace Violet staining (B). Following culture for 8 days, viral production

(C) was measured using the produced HIV-1 virions to infect TZM-bl reporter cells at the indicated time points. The cells were lysed for western blotting to assess the levels of GJB2 and GAPDH (D). Data are presented as the mean ± SD from three triplicates.

Figure S6. Silencing *GJB2* promotes cell-to-cell HIV-1 tranmission.

(A and B) Lentiviral shRNA-transduced MDMs were seeded at 3.5 × 10^5 cells per well and used as target cells in an in vitro infection assay with ACH2 cells as donor cells. ACH2 cells were pretreated with PMA for 24 hours. Co-cultures were established at a 1:1 donor-to-target ratio and maintained for 6 hours, with or without transwells (0.4-μm pore size). To remove ACH2 cells after coculture with MDMs, four extensive PBS washes were performed. At 72 hours post-coculture, Gag-positive MDMs were analyzed by flow cytometry, and (B) western blotting was performed to measure GJB2 and GAPDH levels using their specific antibodies.

Figure S7. GJB2 inhibits HIV-1 spread in DCs.

(A and B) Lentiviral shRNA-transduced MDDCs were treated with VLP-Vpx and infected with 2 ng (low dose) or 20 ng (high dose) of HIV-1_{AD8} for 18 days. Viral production was measured using p24 ELISA at the indicated time points (A). The aliquoted cells were lysed for western blotting to assess the GJB2 and GAPDH levels (B). (C and D) Lentiviral CRISPR-Cas9-transduced MDDCs (with sgRNAs to disrupt *GJB2* loci) were treated with VLP-Vpx and infected with 10 ng of HIV-1_{AD8} for 18 days. Viral production was measured using p24 ELISA at the indicated time points (C). The aliquoted cells were lysed for western blotting to assess the GJB2 and GAPDH levels (D).

Table S1. Backgrounds of PLWH whose CD4$^+$ T cells were isolated and used for *ex vivo* study (related to Fig. S5).

Table S2. Primer sequences.

The primer pairs and sequences used in this study are listed in this table.

Figure S1

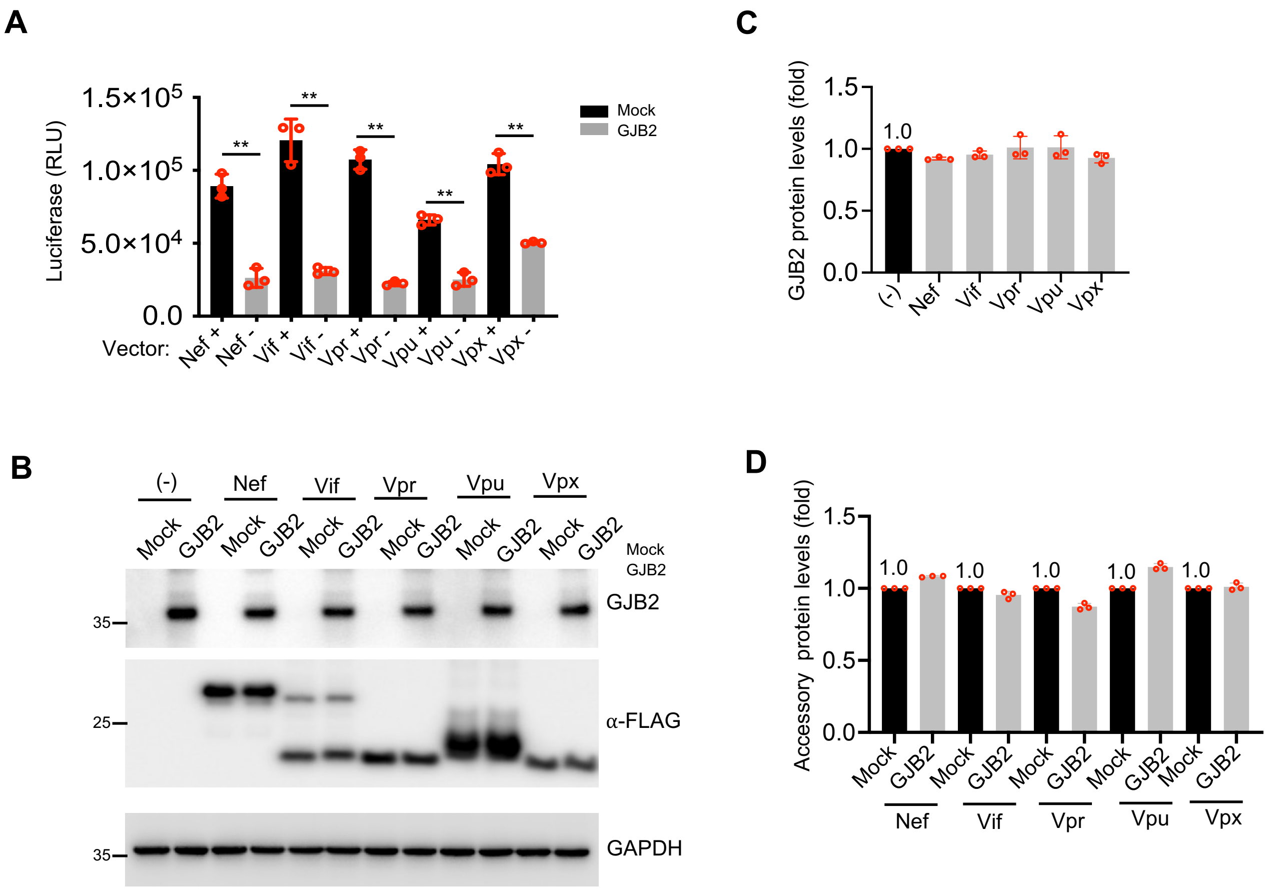

A
Luciferase (RLU)
1.5×10^5
1.0×10^5
5.0×10^4
0.0
Mock
GJB2
**
Vector:
Nef +
Nef -
Vif +
Vif -
Vpr +
Vpr -
Vpu +
Vpu -
Vpx +
Vpx -
B
(-)
Nef
Vif
Vpr
Vpu
Vpx
Mock
GJB2
35
25
GJB2
α-FLAG
GAPDH
C
GJB2 protein levels (fold)
1.5
1.0
0.5
0.0
(-)
Nef
Vif
Vpr
Vpu
Vpx
D
Accessory protein levels (fold)
1.5
1.0
0.5
0.0
Mock
GJB2
Nef
Vif
Vpr
Vpu
Vpx

Figure S2

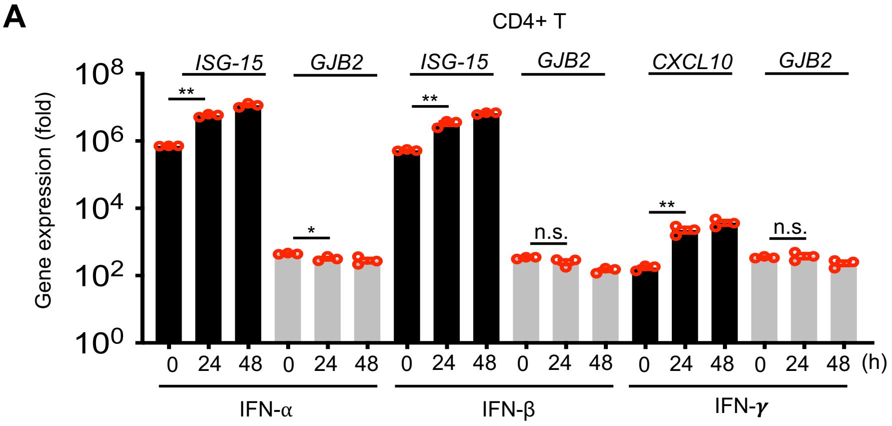
A
CD4+ T
ISG-15
GJB2
ISG-15
GJB2
CXCL10
GJB2
Gene expression (fold)
10^8
10^6
10^4
10^2
10^0
**
*
**
n.s.
**
n.s.
0 24 48 0 24 48 0 24 48 0 24 48 0 24 48 0 24 48 (h)
IFN-α
IFN-β
IFN-γ

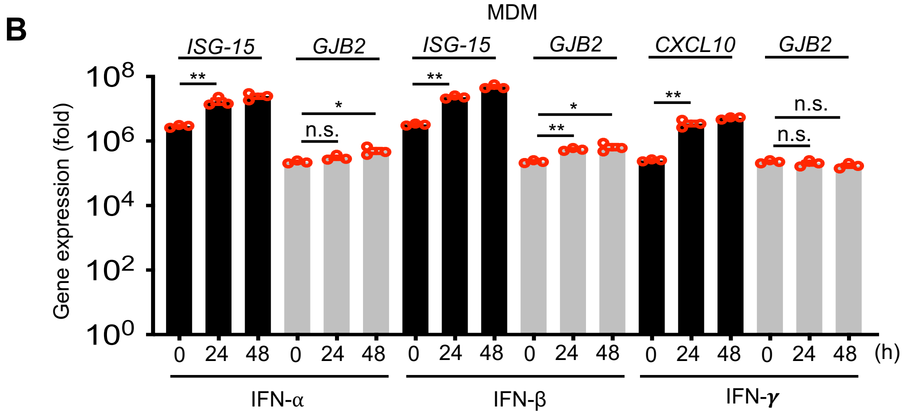
B
MDM
ISG-15
GJB2
ISG-15
GJB2
CXCL10
GJB2
Gene expression (fold)
10^8
10^6
10^4
10^2
10^0
**
*
n.s.
**
*
**
**
n.s.
n.s.
0 24 48 0 24 48 0 24 48 0 24 48 0 24 48 0 24 48 (h)
IFN-α
IFN-β
IFN-γ

Figure S3

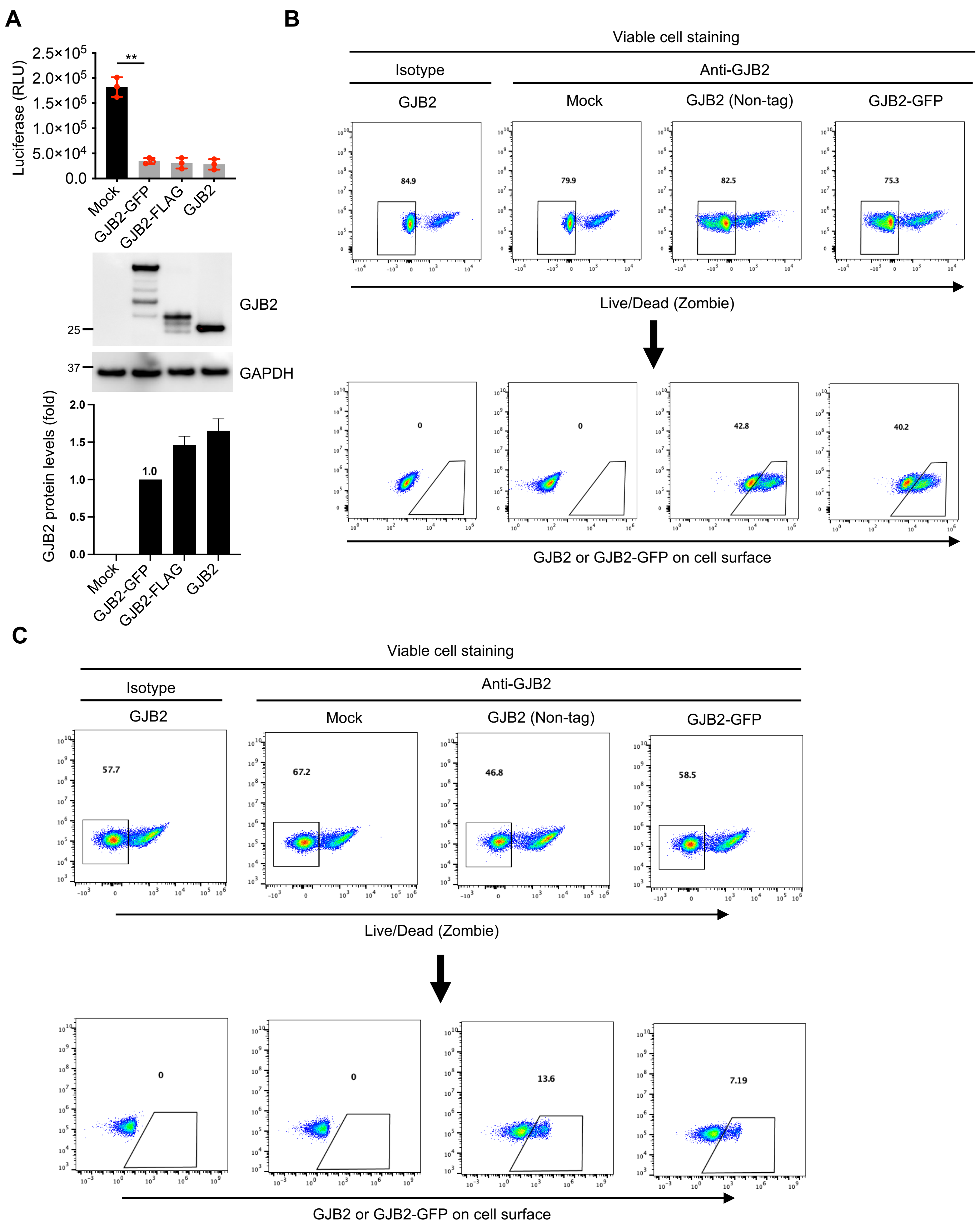
A
B
C
Luciferase (RLU)
**
Mock
GJB2-GFP
GJB2-FLAG
GJB2
25
37
GAPDH
GJB2 protein levels (fold)
1.0
Viable cell staining
Isotype
Anti-GJB2
GJB2 (Non-tag)
GJB2-GFP
84.9
79.9
82.5
75.3
Live/Dead (Zombie)
0
42.8
40.2
GJB2 or GJB2-GFP on cell surface
57.7
67.2
46.8
58.5
13.6
7.19

Figure S3

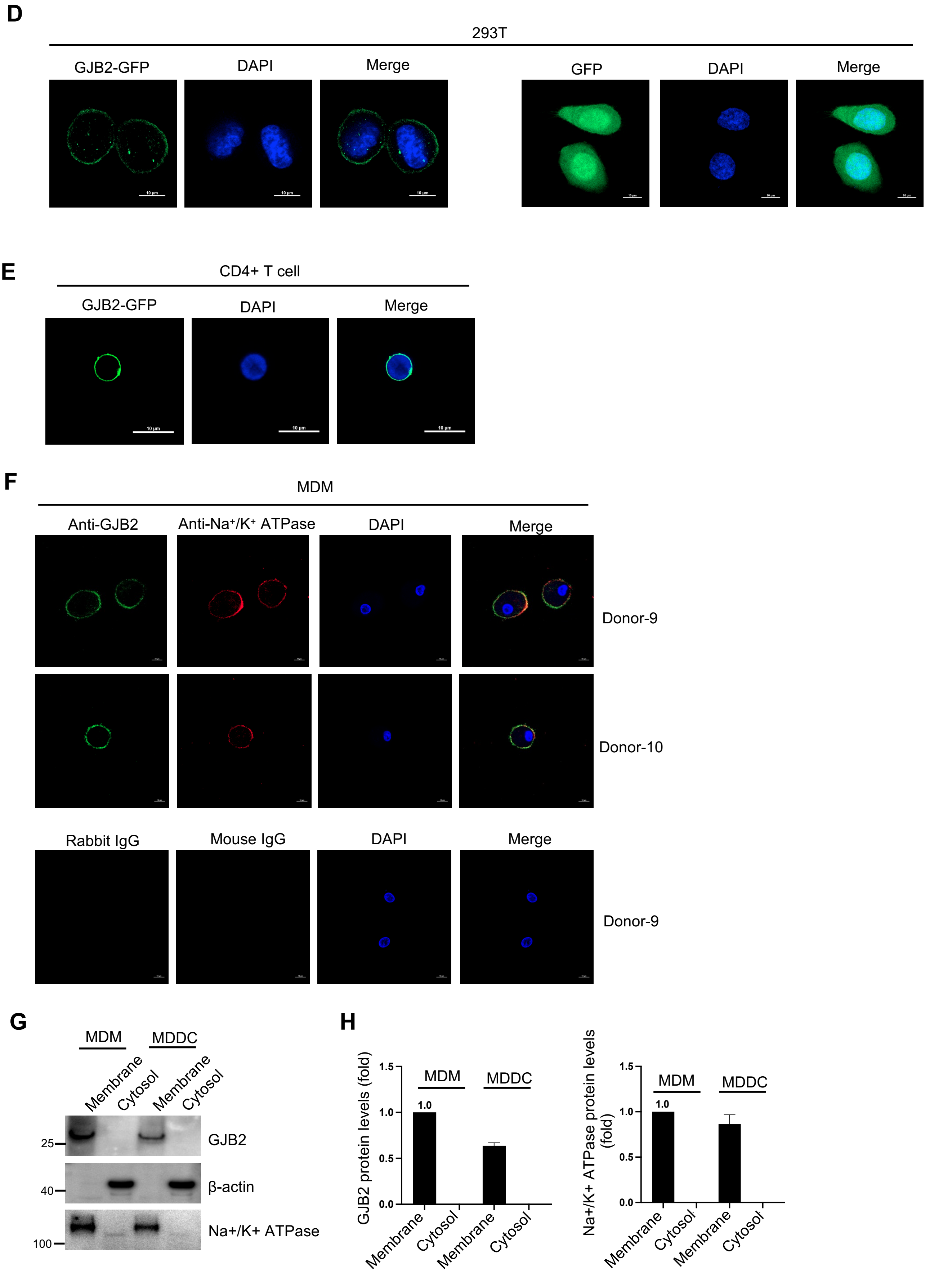
D
293T
GJB2-GFP
DAPI
Merge
GFP
DAPI
Merge
E
CD4+ T cell
GJB2-GFP
DAPI
Merge
10 μm
F
MDM
Anti-GJB2
Anti-Na⁺/K⁺ ATPase
DAPI
Merge
Donor-9
Donor-10
Rabbit IgG
Mouse IgG
DAPI
Merge
Donor-9
G
MDM
MDDC
Membrane
Cytosol
Membrane
Cytosol
25
40
100
GJB2
β-actin
Na+/K+ ATPase
H
GJB2 protein levels (fold)
MDM
MDDC
1.0
1.5
1.0
0.5
0.0
Membrane
Cytosol
Membrane
Cytosol
Na+/K+ ATPase protein levels (fold)
MDM
MDDC
1.0
1.5
1.0
0.5
0.0
Membrane
Cytosol
Membrane
Cytosol

Figure S4

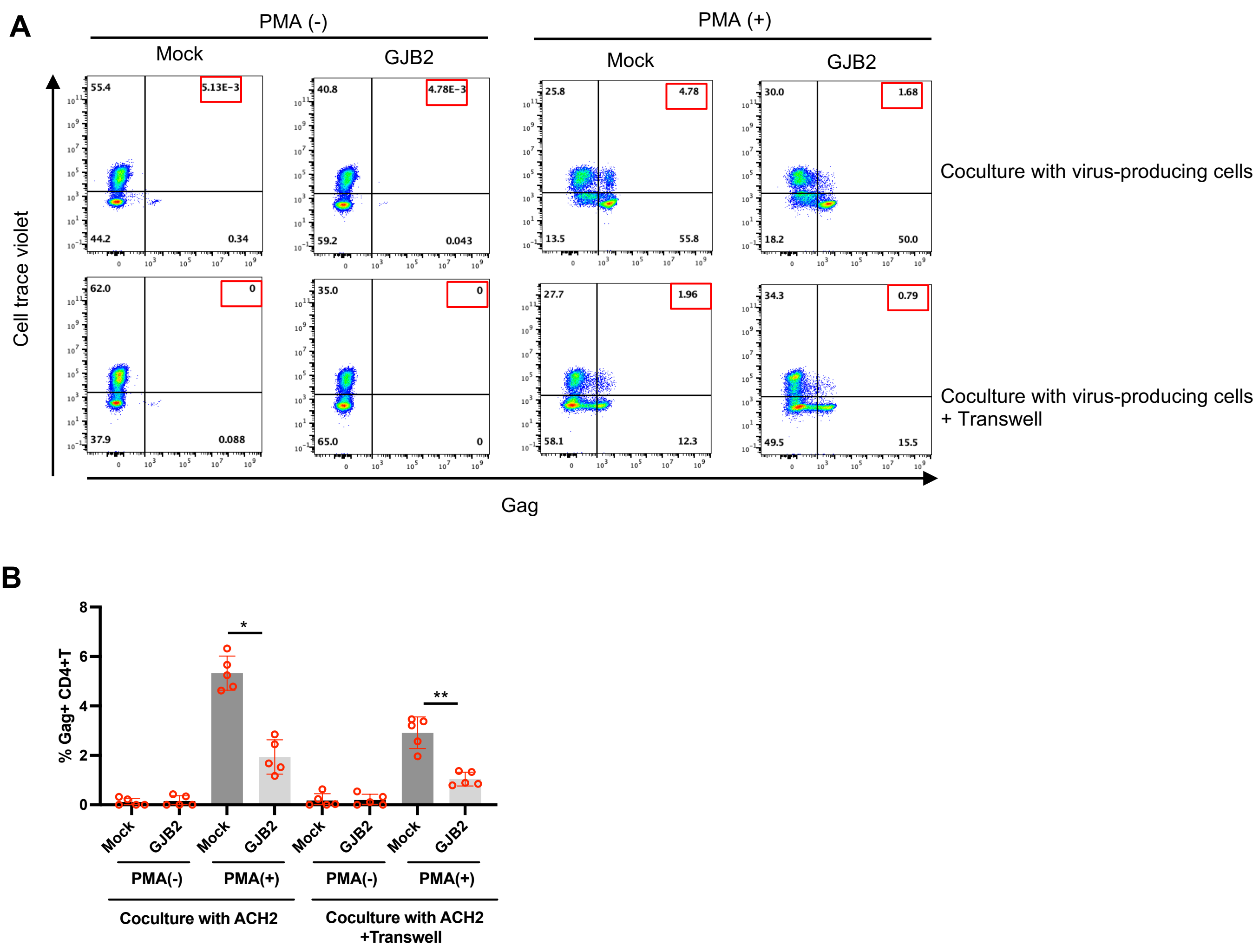

A
PMA (-)
PMA (+)
Mock
GJB2
Mock
GJB2
Cell trace violet
Gag
55.4
5.13E−3
44.2
0.34
40.8
4.78E−3
59.2
0.043
25.8
4.78
13.5
55.8
30.0
1.68
18.2
50.0
62.0
0
37.9
0.088
35.0
0
65.0
0
27.7
1.96
58.1
12.3
34.3
0.79
49.5
15.5
Coculture with virus-producing cells
Coculture with virus-producing cells + Transwell
B
% Gag+ CD4+T
*
**
Mock
GJB2
Mock
GJB2
Mock
GJB2
Mock
GJB2
PMA(-)
PMA(+)
PMA(-)
PMA(+)
Coculture with ACH2
Coculture with ACH2 +Transwell

Figure S5

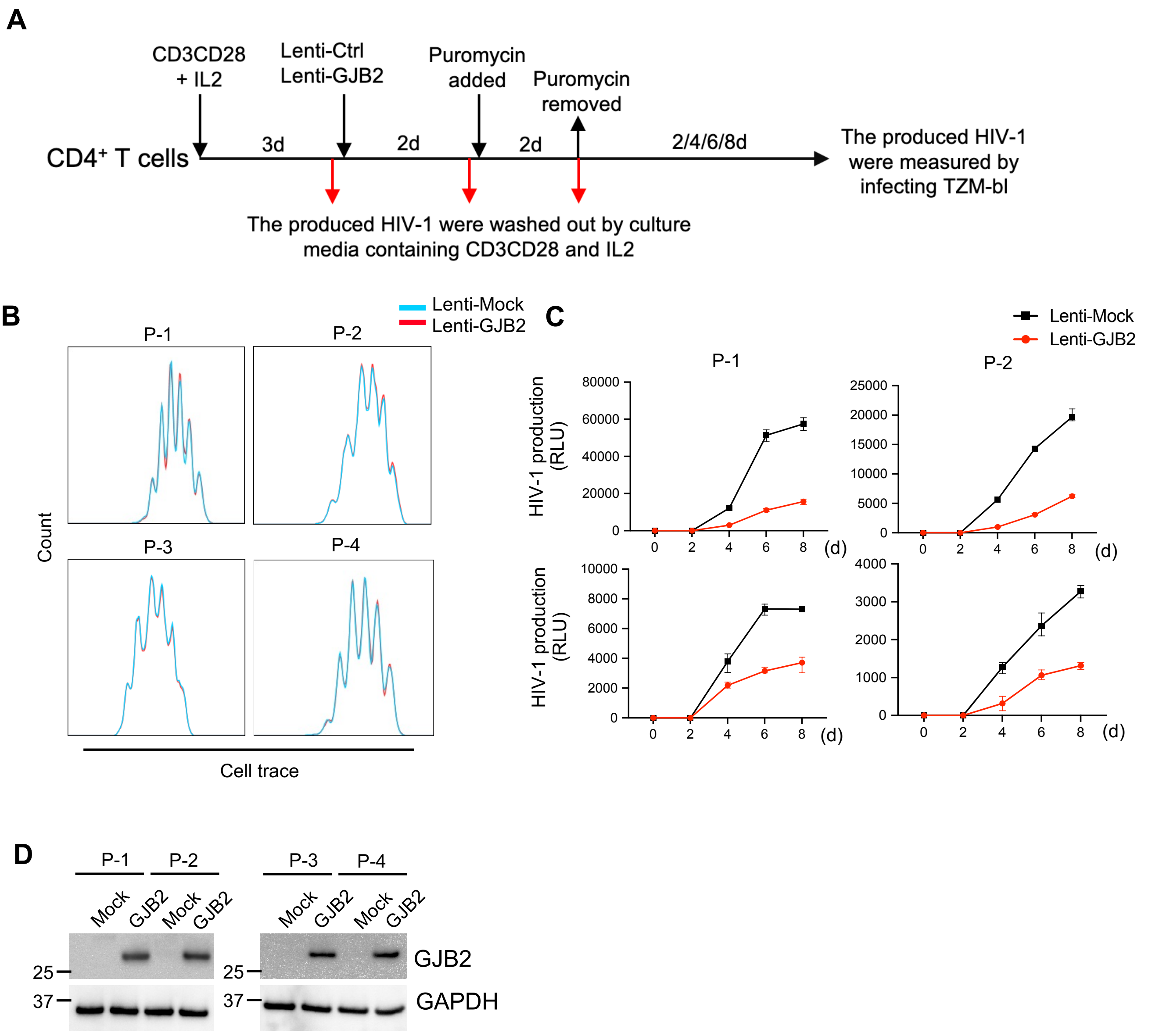
A
CD3CD28 + IL2
Lenti-Ctrl
Lenti-GJB2
Puromycin added
Puromycin removed
CD4⁺ T cells
3d
2d
2d
2/4/6/8d
The produced HIV-1 were measured by infecting TZM-bl
The produced HIV-1 were washed out by culture media containing CD3CD28 and IL2
B
Lenti-Mock
Lenti-GJB2
P-1
P-2
P-3
P-4
Count
Cell trace
C
Lenti-Mock
Lenti-GJB2
P-1
P-2
HIV-1 production (RLU)
80000
60000
40000
20000
0
25000
20000
15000
10000
5000
10000
8000
6000
4000
2000
3000
1000
0 2 4 6 8 (d)
D
P-1
P-2
P-3
P-4
Mock
GJB2
25
37
GJB2
GAPDH

Figure S6

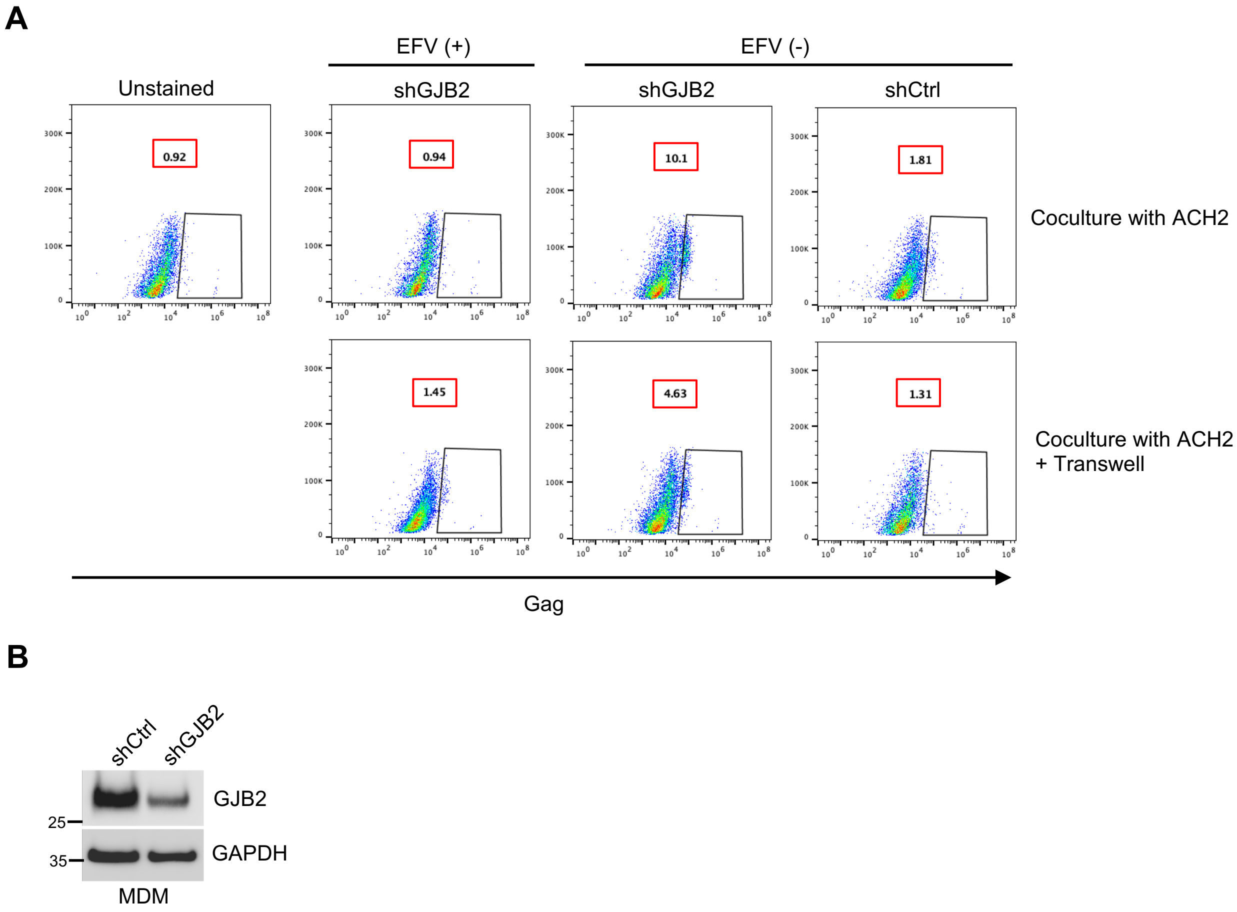

A
EFV (+)
EFV (-)
Unstained
shGJB2
shGJB2
shCtrl
0.92
0.94
10.1
1.81
Coculture with ACH2
1.45
4.63
1.31
Coculture with ACH2
+ Transwell
Gag
B
shCtrl
shGJB2
GJB2
25
35
GAPDH
MDM

Figure S7

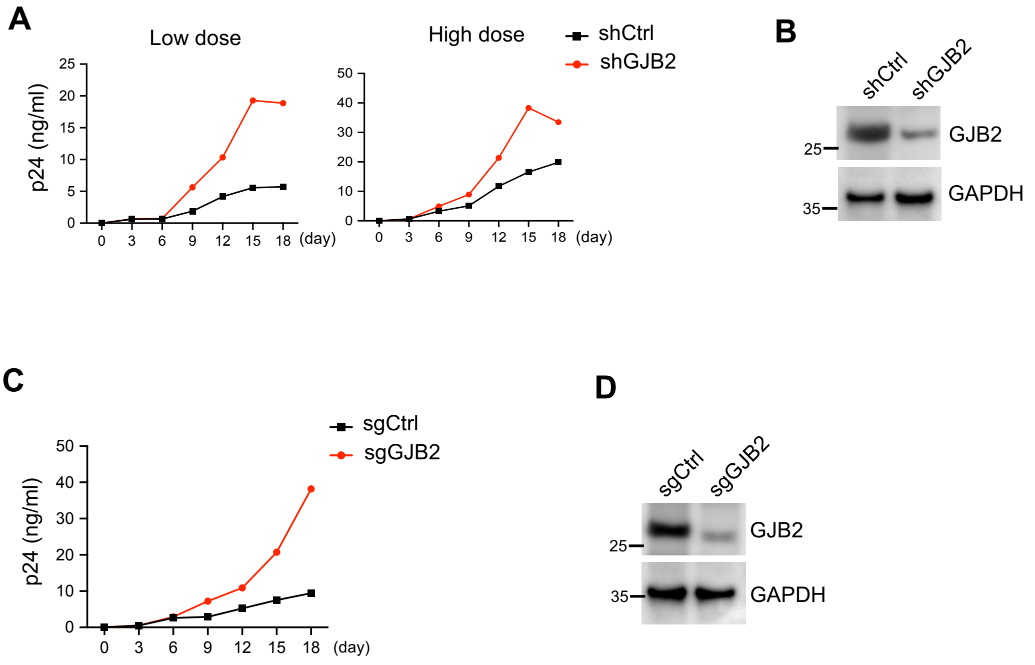
A
Low dose
High dose
shCtrl
shGJB2
p24 (ng/ml)
(day)
B
shCtrl
shGJB2
GJB2
GAPDH
25
35
C
sgCtrl
sgGJB2
p24 (ng/ml)
(day)
D
sgCtrl
sgGJB2
GJB2
GAPDH
25
35

Table S1. Patients' backgrounds for ex vivo study, related to Fig. S5.

PID	CD4 (cells/μL)	VL (copies/mL)	Sex	Age
1	550	target not detected	Male	37
2	469	target not detected	Male	31
3	623	target not detected	Male	31
4	689	target not detected	Male	42

Table S2

Gene	Sequences
GAPDH for	5′- AATGACCCCTTCATTGAC
GAPDH rev	5′-TCCACGACGTACTCAGCGC
GJB2 for	5′-TCGCATTATGATCCTCGTTGTG
GJB2 rev	5′-GGGGAAGTAGTGATCGTAGCAC
ISG-15 for	5′-CGCAGATCACCCAGAAGATCG
ISG-15 rev	5′-TTCGTCGCATTTGTCCACCA
CXCL10 for	5′-GTGGCATTCAAGGAGTACCTC
CXCL10 rev	5′-TGATGGCCTTCGATTCTGGATT
